# Platelet-rich plasma enhances local homing of umbilical cord-derived mesenchymal stem cells to articular cartilage by increasing the quantity and activation of integrin ꞵ1

**DOI:** 10.1186/s13287-025-04593-y

**Published:** 2025-09-02

**Authors:** Min Ji Lee, Soo Hyun Kim, Sue Shin, Tae Woo Kim, Chris Hyunchul Jo

**Affiliations:** 1 Department of Translational Medicine, Seoul National University College of Medicine, 103 Daehak-ro, Jongno-gu, Seoul, 03080 Korea; 2 Department of Orthopedic Surgery, SMG-SNU Boramae Medical Center, Seoul National University College of Medicine, 20 Boramae-ro 5-gil, Dongjak-gu, Seoul, 07061 Korea; 3 Department of Laboratory Medicine, SMG-SNU Boramae Medical Center, Seoul National University College of Medicine, 20 Boramae-ro 5-gil, Dongjak-gu, Seoul, 07061 Korea

**Keywords:** Cartilage regeneration, Mesenchymal stem cells, Homing, Engraftment

## Abstract

**Background:**

Enhancing mesenchymal stem cells (MSCs) engraftment at the degenerative cartilage is important to increase the therapeutic effect of cartilage regeneration. Platelet-rich plasma (PRP) is known to have anti-inflammatory and anabolic effects for the treatment of osteoarthritis and has been reported to be commonly used with MSCs. However, little is known about the effects of PRP on MSCs adhesion to cartilage extracellular matrix (ECM). The purpose of this study was to investigate how PRP pre-conditioning enhances MSC adhesion to cartilage ECM and improves the efficacy of MSCs in promoting cartilage regeneration in a rat osteochondral defect model.

**Method:**

*In vitro *adhesion of umbilical cord-derived MSCs (UC MSCs) to collagens, fibronectin, and hyaluronic acid and to osteochondral explants was measured with or without PRP pre-conditioning using a cell viability assay. The mRNA expression of integrin subunits and the activity level of integrin ꞵ1 (ITGB1) were evaluated using RT-PCR and western blot, respectively. After establishing an osteochondral defect in the femoral trochlea in Sprague-Dawley rats (*n* = 26), UC MSCs with PRP were locally injected into the defect. After 4 weeks, macroscopic and histological evaluations were performed using ICRS and O’Driscoll scoring systems.

**Results:**

PRP significantly increased the adhesion of MSCs to collagens, fibronectin, and degenerative osteochondral explants, and this effect was inhibited primarily by ITGB1. Pre-conditioning of MSCs with PRP altered ITGB1 into an active form without cell-to-ECM interaction. Pre-conditioning UC MSCs and type II collagen with PRP prior to adhesion significantly enhanced their adhesion by 4.1-fold compared to the control. In histological evaluation, the O’Driscoll scores of UC MSCs (11.56 ± 0.53, *p* < .001) and UC MSCs + PRP group (14.13 ± 0.53, *p* < .001) were significantly higher than the control group (4.40 ± 0.82). PRP as a vehicle of MSCs showed a higher O’Driscoll score than MSCs with saline by 1.2-fold (*p* = .035).

**Conclusion:**

This suggests that pre-conditioning MSCs and cartilage ECM with PRP synergistically enhanced MSC adhesion to the cartilage ECM by increasing both the quantity and activation of ITGB1 in MSCs, thereby enhancing the therapeutic effects of MSCs on articular cartilage regeneration in a rat osteochondral defect model.

**Graphical abstract:**

**Figure:**
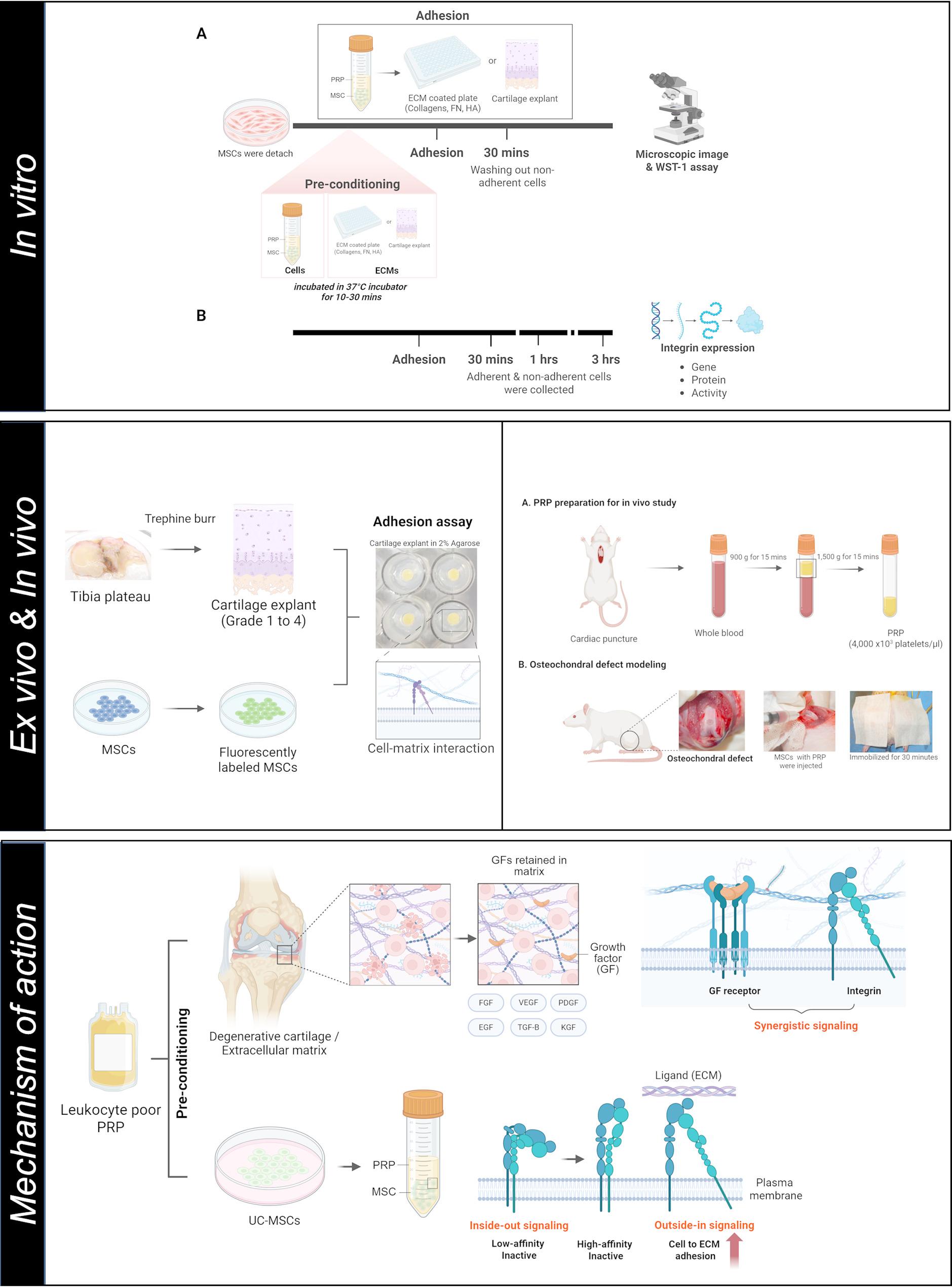
Graphical abstract demonstrated that treating PRP on UC MSCs and ECM enhances cell-to-ECM adhesion via activating beta 1 integrin

**Supplementary Information:**

The online version contains supplementary material available at 10.1186/s13287-025-04593-y.

## Background

Articular cartilage has a limited regenerative potential due to its avascularity and low cellularity [1]. If left untreated, destruction of articular cartilage usually progresses to the degeneration of the articular surface, or early osteoarthritis (OA), and then further develops to advanced osteoarthritis which accompanies cartilage loss, pain, stiffness, and joint disability [2–4]. Current treatments for OA, including microfracture, osteoarticular autograft transfer system, and mosaicplasty have limitations in forming fibrocartilage rather than hyaline cartilage [5]. Positive results for autologous chondrocyte implantation (ACI) have been reported, however, obtaining a sufficient number of chondrocytes from a patient is still challenging [6]. To overcome these limitations, transplanting mesenchymal stem cells (MSC) into the damaged cartilage site has been developed to prevent progressive degeneration and OA [6, 7].

Mesenchymal stem cells (MSCs) show therapeutic potential in their immunomodulatory and regenerative potentials to early- to middle-stage OA [8]. The paracrine effects of MSCs contribute to regeneration with anti-inflammatory and chondroprotective effects [9]. Several clinical studies have confirmed the safety and efficacy of MSC therapies for cartilage defects [10]. However, preclinical and clinical studies show inconsistent data on regenerating cartilage tissue [11]. A possible explanation for these results could be the small fraction of adherent cells to its target tissue and their viability in the joint microenvironment. In a hostile and inflammatory microenvironment in the joint cavity, initial adherent capability to target tissue is important since their sufficient interaction with the matrix supports their viability via reducing “anoikis” [12, 13]. Therefore, increasing the viability and retention of MSCs into the damaged cartilage is a key factor in determining the therapeutic effect of cartilage regeneration [14]. MSCs derived from the umbilical cord have gained significant attention due to their easy and non-invasive isolation from medical waste after delivery, making them a cost-effective source for allogeneic MSC therapy. Our previous studies have demonstrated their strong proliferative and self-renewal capacities, as well as their regenerative effects on tendon repair [15–19]. Many vehicles, such as hyaluronic acid, fibrin glue, biomaterials, and biofactors, have been developed to improve the delivery system of MSCs to its damaged tissue [6]. Modification of cell surface that leads to the expression of particular receptors on the surface of MSCs with specific binding affinity to the ligands on the target sites using an antibody, peptide, genetics, or selectin was also studied [20, 21]. Nonetheless, to our current knowledge, no satisfactory strategy has been found [8].

Articular cartilage damage may initially be confined to the cartilage layer, but further degeneration can lead to subchondral bone exposure [22, 23]. Major extracellular matrix proteins that are composed of cartilage are collagen II, collagen XXII, COMP, and fibronectin, whereas collagen I is a major extracellular matrix protein in bone. Integrins are transmembrane receptors that play critical roles in cell-matrix interaction [24]. Among these proteins, only collagen I, collagen II, and fibronectin are reported to mediate MSC adhesion mainly via the integrin beta 1 subunit [25, 26]. Using natural and synthetic cell scaffolds showed high cell engraftment efficiency, but requiring surgical intervention is a limitation in clinical use. Few studies have tried to enhance the expression of adhesion and migration receptors in terms of cartilage cell therapy. Previous reports have demonstrated that an appropriate concentration of magnesium has a stimulatory effect on integrin binding of MSCs to collagen, whereas no improvement in cartilage adhesion of MSCs was shown with some inflammatory factors [11, 25, 27, 28].

The hostile microenvironment of damaged articular cartilage with inflammation, hypoxia, and insufficient blood supply is a critical factor that could lead to the low survival rate of injected cells. Pre-conditioning (PreCd) of the joint cavity with an appropriate agent, which alleviates the inflammatory condition, could enhance the ability of the injected MSCs to resist adverse microenvironments [29]. Enhancing MSC capability of adhesion to its target and improving the adverse microenvironment of cartilage are both important to prolong the survival rate of MSCs, therefore increasing the therapeutic effects of intra-articular injection of MSCs [11]. Platelet-rich plasma (PRP) is a widely used natural reservoir of cytokines and growth factors in musculoskeletal diseases. PRP has been shown to have anti-inflammatory effects and to stimulate the healing process [30, 31]. Current clinical outcomes explained PRP can be expected to improve knee OA by attenuating pain and improving joint function when compared with hyaluronic acid or corticosteroid [32, 33]. PRP showed its potential to fill cartilage defects in animal models and suppress inflammatory mediators in cartilage explants [34, 35]. Nonetheless, very few studies investigated the effects of PRP on MSCs adhesion to cartilage defects [11, 14]. In this study, we aimed to investigate how PRP pre-conditioning enhances MSC adhesion to cartilage ECM and improves the efficacy of MSCs in promoting cartilage regeneration in a rat osteochondral defect model.

## Methods

### Preparation of osteoarthritic synovial fluids (OA SF)

This study was approved by our Institutional Review Board (SMG-SNU BMC 30-2020-039). Synovial fluid samples were obtained from patients (*n* = 26; 73.8 ± 6.2 years; 6 males and 20 females) with severe osteoarthritis, classified as Kellgren-Lawrence grade 4, who were undergoing total knee replacement. Synovial fluid samples were centrifuged at 4 °C and 1,000 g for 15 min to remove cell debris, and the supernatants were stored at -80 °C until required. For the experiment, synovial fluid from 3 donors was pooled and used.

### Preparation and characterization of allogeneic PRP

Allogeneic leukocyte-poor PRP (*n* = 7; 56.1 ± 12.7 years; 3 males and 4 females) was prepared using a plateletpheresis system with a leukoreduction set (COBE Spectra LRS Turbo, Lakewood, CO, USA) from patients undergoing arthroscopic rotator cuff repair who were otherwise healthy. Safety of the PRP was confirmed negative for hepatitis B (HBV), hepatitis C (HCV), human immunodeficiency virus (HIV), and syphilis (VDRL). Platelets in PRP were diluted to around 1,000 × 10^3^ platelets/µL. Complete blood counts of PRPs were measured using a fully automated analyzer (XE-2100, Sysmex Corp, Kobe, Japan) and the concentrations of fibrinogen were counted by an automated coagulation analyzer (CA-7000, Sysmex Corp). 10% of calcium gluconate (Calcium Gluconate Inj, Daihan, Korea) was used to activate platelets for an hour, and the supernatant was used in the study.

### Isolation and culture of UC MSC

Human umbilical cords were obtained from healthy full-term deliveries by cesarean section, and UC MSCs (*n* = 4, 33.5 ± 7.5 years) were isolated and cultured using the previously reported method [16] UC-MSCs were cultured with low-glucose Dulbecco’s modified Eagle medium (LG-DME; Hyclone, Logan, USA) supplemented with 10% fetal bovine serum (FBS; Hyclone) and 1% antibiotic-antimycotic solution (100 U/mL penicillin, 100 µg/mL streptomycin, and 0.25 µg/mL amphotericin B; Welgene). UC-MSCs were cryopreserved at passage 8, thawed, and cultured until passage 9–10 for experiments. UC-MSCs were confirmed with high proliferative capacity and resistance to aging markers up to passage 10, maintaining phenotypic stemness and stable genomic integrity in our previous study [36, 37]. When the cells reached approximately 80% confluence, they were detached and used for subsequent experiments.

### *In vitro* cell adhesion assay

Culture plate was coated with 10 µg/ml Type 1 collagen (Corning, USA), 1 µg/ml type 2 collagen (Biosciences, USA), 5 µg/ml fibronectin (Biosciences, USA), or 5 mg/ml hyaluronic acid (Hyruan Plus Injection, LG life Sciences, Korea) by immobilizing overnight at 4 °C. Non-specific binding was blocked with 1% bovine serum albumin (BSA; Sigma) in DPBS for 90 min at 37 °C. UC-MSCs (1 × 10⁵ cells/cm²) were resuspended either in 100% (v/v) PRP or in saline, seeded onto pre-coated plates, and incubated at 37 °C for 30 min. For pre-conditioning of UC-MSCs, cells were resuspended with or without 100% PRP for 10, 20, or 30 min prior to seeding onto ECM-coated plates. For ECM pre-conditioning, ECM-coated plates were incubated with 10%, 50%, or 100% PRP for 30 min, after which UC-MSCs were seeded and allowed to adhere for an additional 30 min. Non-adherent cells were washed with DPBS three times. The numbers of adherent cells were measured with a cell viability assay kit (EZ-CYTOX; daeillab, Korea), and the optical densities of the microplate wells were measured with a microplate reader (SpectraMax Plus384; Molecular Devices, Sunnyvale, California). For neutralizing integrin receptors, UC MSCs were incubated with the antibody at 37 °C for 30 min, and then used for adhesion (see supplementary Table 2). To observe gene and protein expression, UC MSCs were allowed to adhere to the ECM for 30 min, 1 h, and 3 h. Both adherent and non-adherent cells were collected and analyzed to assess their expression and activity following cell adhesion.

### *Ex vivo* cell adhesion assay

Tibia plateaus were obtained from the patients undergoing total knee replacement. Osteochondral bones (*n* = 35; 72.1 ± 7.3 years; 11 males and 24 females) were prepared using a 4 mm-diameter burr. The degree of degeneration of articular cartilage was measured according to a slightly modified Yulish and Outerbridge classification (Supplementary Table 3) [38, 39]. G1, cartilage surface softening without morphologic defect; G2, partial-thickness defect less than 50% of the cartilage thickness; G3, partial-thickness defect from 50 to 100% of the cartilage thickness; G4, Complete loss with exposure of subchondral bone. The osteochondral bones were embedded in 2% agarose in 48 well plate and washed with DPBS (containing 1% antibiotic-antimycotic solution) to remove any remaining debris. UC-MSCs (2 × 10⁵ cells/cm²) were labeled with Calcein-AM (Sigma, USA), seeded onto the osteochondral bone surface, and incubated at 37 °C for 30 min to allow immobilization. Pre-conditioning of the cells and ECM was performed as described in the previously described method. Non-adherent cells were washed out with DPBS for third. Adherent cells were imaged using fluorescence microscopy (Leica DMI 4000B, Leica, Wetzlar, Germany) and measured with a cell viability assay kit as mentioned above.

### Semi-quantitative RT-PCR analysis

Total RNA was extracted from UC-MSCs using the eCube Tissue RNA Mini Kit (PhileKorea, Korea), and RNA concentration was quantified using a NanoDrop 2000 spectrophotometer (NanoDrop, Wilmington, Delaware). RNA purity (A260/A280 ratio) values between 1.8 and 2.0 were considered acceptable for further use. First-strand complementary DNA (cDNA) was synthesized using HiSenScript™ RH(-) RT Premix kit (iNtRON, Sungnam, Korea). An equal amount of the synthesized cDNA was used for RT-PCR using Maxime™ PCR PreMix (i-StarTaq, iNtRON). Primers used in the study were listed in supplementary Table 4. Data were normalized to glyceraldehyde 3-phospate dehydrogenase (GAPDH).

### Western blotting

Both adherent cells and non-adherent cells were collected and washed with DPBS for twice, and lysed using PRO-PREP™ protein extraction solution (iNtRON). The protein concentration of protein lysates was measured using the Pierce™ BCA Protein Assay Kit (Thermo Scientific, USA), and 10 µg of protein was electrophoresed into 8% SDS-PAGE gels. A PVDF membrane with 0.45 μm pore size was used to blot, and 5% skim milk in Tris-buffered saline with 0.1% tween 20 was used to block. The following primary antibodies were diluted in TBS-T and incubated overnight at 4℃: Integrin beta 1/CD29 antibody (ATGA0485, ATGen, Korea), activated-beta 1-integrin conformation antibody (MAB2079Z, Sigma, USA), β-actin antibody (sc47778, Santacruz Bio). Following three washes in TBS-T, HRP-conjugated secondary antibody (SA001, Gendpot, USA) was diluted and incubated for 45 min. The membrane was scanned using ImageQuant LAS4000 mini (GE Healthcare Life Sciences, Little Chalfont, UK). Non-reducing conditions were used to prepare the lysates for activated β1-integrins [40]. Densitometric quantifications were analyzed using Image J software (National Institutes of Health, Bethesda, MD).

### Immunocytochemical analysis

Chamber slides (154941, Thermo Scientific, USA) were pre-coated with type I collagen, type II collagen, and fibronectin as described above. Non-adherent cells were removed by washing three times with DPBS at 10, 20, and 30 min. The remaining cells were fixed with 4% paraformaldehyde (PFA) and blocked with 3% BSA. The primary antibody was diluted in 3% BSA (1:200) and incubated overnight at 4 °C. The slides were stained with Alexa Fluor 488 (Invitrogen, USA) for 45 min at room temperature (RT), followed by washing with DPBS three times. The details of antibodies used in the study are listed in Supplementary Table 2. The slides were mounted using Vectashield containing DAPI (Vector Laboratories Inc., Burlingame, CA).

### *In vivo* animal experiment

The work has been reported in line with the ARRIVE guidelines 2.0. Animal procedures were conducted in accordance with the protocol approved by the Seoul Metropolitan Government Seoul National University Boramae Medical Center Institutional Animal Care and Use Committee (IACUC_2017-0018). This study was carried out strictly following the guidelines for the IACUC and IRB. Anesthesia was induced using Zoletil (a combination of 145.5 mg of tiletamine hydrochloride and 140.9 mg of zolazepam hydrochloride per 5 mL vial) and Rompun (xylazine) at doses of 30 mg/kg and 10 mg/kg, respectively. An Osteochondral defect with a diameter of 2 mm and a depth of 2 mm was established in the femoral trochlea using a trephine burr. Sprague-Dawley male rats (12 weeks old, 340 ~ 360 g) were randomly divided into three groups: (1) saline group (*n* = 6) (2), UC-MSCs group (*n* = 10) (3), UC-MSCs + PRP group (*n* = 10). The animals were housed under controlled conditions (22 ± 2 °C, 50 ± 10% humidity, 10–20 air changes per hour, 12-hour light/dark cycle, and light intensity of 150–300 lx) in poly sulfonic acid cages (W 395 x L 346 x H 213 mm), with two rats per cage during acclimatization, quarantine, and the experimental period. For PRP preparation, 10 mL of whole blood was collected from Sprague-Dawley rats and centrifuged at 900 g for 15 min at 22 °C. PRP was concentrated by further centrifugation at 1500 g for 15 min as previously described [41]. Platelet counts were adjusted to 4000 × 10³ platelets/µL using platelet-poor plasma. Complete blood counts and fibrinogen levels were measured with an automated analyzer. MSCs with or without PRP were implanted in the defect and immobilized for 30 min. Body weight was measured at the time of sacrifice, and no significant differences in body weight were observed between the groups, indicating that there were no major health complications associated with the treatments. After 4 weeks, rats were sacrificed in a carbon dioxide chamber (60 ~ 70% CO_2_) for further procedure.

### Histological evaluation

For cartilage explants, each grade (G1 to G4) of osteochondral bones was fixed with 4% PFA for two days at 4 ℃ and decalcified with 5% formic acid (Biosesang, Korea) for 3 days. After dehydrating with a gradient ethanol series, the osteochondral bones were embedded in paraffin blocks. Serial Sect. (4-µm thick) were obtained from the center of the bone and were stained with hematoxylin and eosin (H&E) or safranin-O/fast green (Saf-O). The slides were imaged with light microscopy (U-TVO 63XC; Olympus Corp., Japan). For the animal study, the gross appearance of the defect sites was photographed and scored using the International Cartilage Repair Society (ICRS) macroscopic scoring system [42]. Articular cartilages were fixed in 4% (w/v) paraformaldehyde (PFA; Merck, Darmstadt, Germany) for 48 h, followed by decalcification in 10% EDTA (Sigma-Aldrich, St Louis, MO, USA) for 3 days. Decalcified tissues were dehydrated through an increasing series of ethanol gradients, then embedded in paraffin blocks. The embedded tissues were coronally cut into 4-µm-thick serial sections, and a randomly selected slide was stained with H&E and Saf-O. The slides were blindly scored using the O’Driscoll scoring system, semi-quantified by visual assessment [43].

### Statistical analysis

All data are shown as mean ± standard error. Normality of the data was confirmed using the Shapiro-Wilk test. In vitro and in vivo data were analyzed with one-way analysis of variance (ANOVA) with post hoc analysis using Bonferroni multiple comparison test. For in vitro and ex vivo experiments, biological replicates were performed using at least two independent samples, and technical replicates were conducted with triplicates for each sample. The significance between the means of PRP and PRP pre-conditioning group was analyzed by paired t-test. All statistical analyses were performed with SPSS software version 23 (IBM). Differences of *p* < .05 were considered statistically significant.

## Results

### PRP enhances UC MSC adhesion to articular cartilage depending on the degree of degeneration

**Fig. 1:**
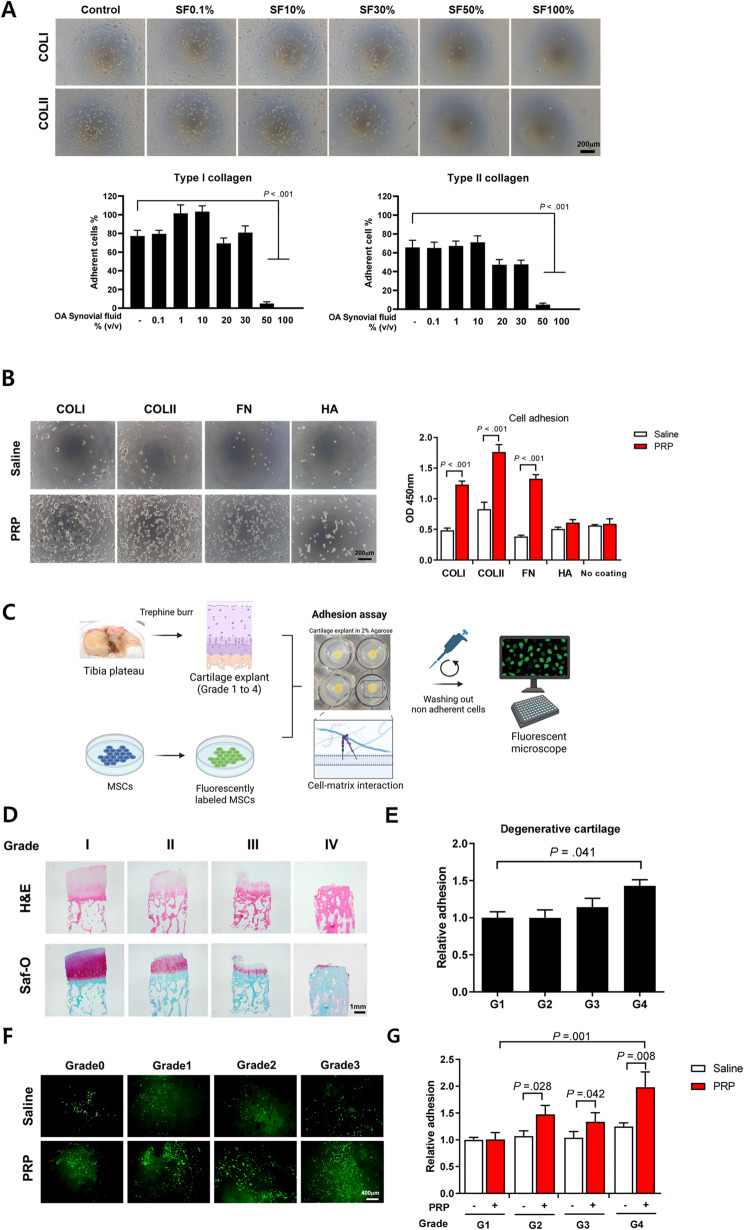
Effects of PRP on UC MSCs adherence to extracellular matrix (ECM). UC MSCs were seeded on a pre-coated plate and incubated at 37 °C for 30 min. (**A**) MSCs showed a dose-dependent loss of adhesion to type I and II collagens in OA synovial fluid diluted with serum-free LG-DMEM, with adhesion reduced by 50–100% (*n* = 12). (**B**) Microscopic images of adherent UC MSCs on ECM proteins consisting of cartilage and bone, and the relative adherence of UC MSCs on ECM were measured using a cell viability assay (*n* = 12). (**C**) A schematic image for the ex vivo adhesion assay using osteochondral blocks. (**D**) Osteochondral blocks were graded by degree of degeneration according to the modified Yulish and Outerbridge classification. (**E**) Adhesion of UC MSCs on cartilage explants of grade 1 to grade 4 (G1 to G4) was measured using a cell viability assay (*n* = 6). (**F**, **G**) Fluorescent images of the top side of cartilage explants with adherent calcein AM-stained UC MSCs. The adherent UC MSCs were measured using a cell viability assay (*n* = 16). All sbar charts represent mean ± standard error

The mean concentrations of platelets, red blood cells, white blood cells, and fibrinogen are shown in Table 1. To confirm the challenges MSCs face in adhering to target ECMs within a cartilage microenvironment, synovial fluid (OA SF) from patients with severe osteoarthritis was utilized. The percentages of adherent cells were then measured (Fig. 1A). Under OA SF conditions ranging from 0 to 30%, UC MSCs showed 69.5–103.4% adhesion to type I collagen, and 47.4–71.1% adhesion to type II collagen. However, with OA SF concentrations between 50 and 100%, MSCs significantly lost their adhesion to both type I and type II collagen. These results highlight the difficulties MSCs encounter in adhering to ECMs in clinical situations where knee OA SF is abundantly present. Given the challenges MSCs face in adhering to ECMs under synovial fluid conditions, we next sought to determine whether PRP could enhance MSC adhesion to key extracellular matrix proteins commonly found in cartilage and bone, such as collagen I, collagen II, fibronectin, and hyaluronic acid. In PRP group, there was a significant increase in the number of cells attached to collagen I, collagen II, and fibronectin (Fig. 1B). The degenerative level of osteochondral bones was classified from grade 1 to grade 4 (Fig. 1D). The number of adherent MSCs to osteochondral bone increased with the severity of damage, and there was a significant 1.43-fold increase in the number of MSCs attached to grade 4 osteochondral bone compared to grade 1 (Fig. 1E). Compared to the control group, MSCs treated with PRP showed a significant increase in adhesion to G2 (1.38-fold; *P* = .028), G3 (1.29-fold; *P* = .042), and G4 (1.59-fold; *P* = .008) cartilage (Fig. 1G).

**Table 1:** Characteristics of PRP used. The mean concentrations of platelets, red blood cells, white blood cells, and fibrinogen. Data are presented as mean ± sd. PPP, platelet-poor plasma; PRP, platelet-rich plasma; RBC, red blood cell; WBC, white blood cell

Characteristics of PRPs used				
Counts of platelets, RBCs, and WBCs; concentration of fibrinogen				
	Platelets,	RBCs,	WBCs,	Fibrinogen,
	×10^6^/µL	×10^6^/µL	×10^6^/µL	mg/dL
Whole blood	171.18 ± 26.16	4.44 ± 0.34	5.84 ± 0.89	245.41 ± 78.10
PPP	4.00 ± 2.00	0.003 ± 0.005	0.02 ± 0.02	167.70 ± 38.02
PRP	1309.86 ± 318.60	0.20 ± 0.09	0.02 ± 0.01	206.19 ± 40.95

### Adhesion of UC MSCs to ECMs mainly through beta 1 integrin

**Fig. 2:**
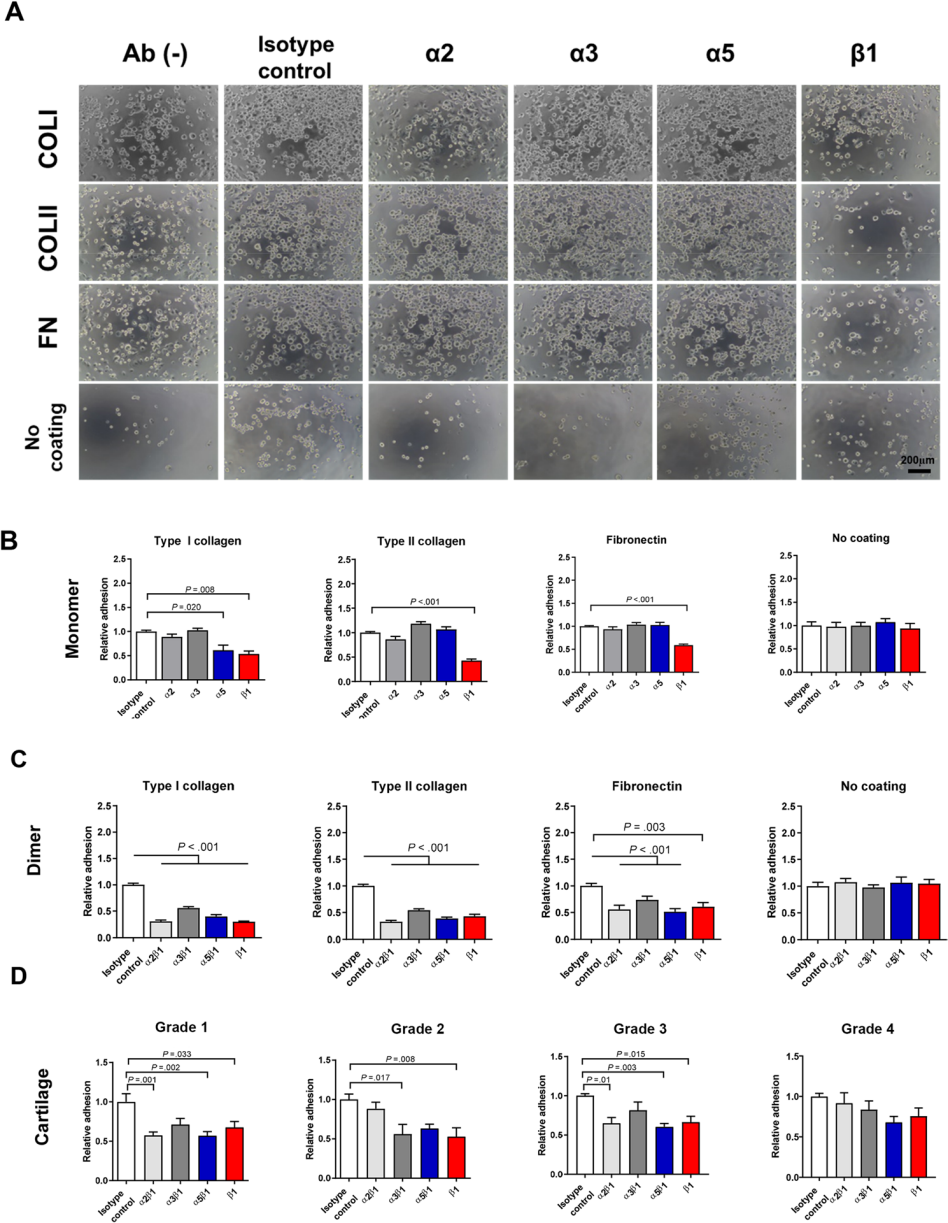
Contribution of individual subunit or heterodimer integrin to UC MSCs’ adhesion to ECM with the PRP treatment. Integrins were neutralized with antibody for 30 min, then UC MSCs were seeded on pre-coated plates and incubated at 37 °C for 30 min for cell adhesion (*n* = 9). (**A**) Microscopic images of adherent UC MSCs with neutralizing antibodies. (**B**) Each individual subunit of integrin was functionally blocked with a neutralizing antibody, and their adhesion to ECM proteins was measured using a cell viability assay. (**C**) Heterodimer integrins were functionally blocked with neutralizing antibody, and their adhesion to ECM proteins was measured using a cell viability assay. (**D**) Heterodimer integrin was neutralized with antibodies, and their adhesion to G1 to G4 cartilage explants was measured using a cell viability assay. All bar charts represent mean ± standard error

**Fig. 3:**
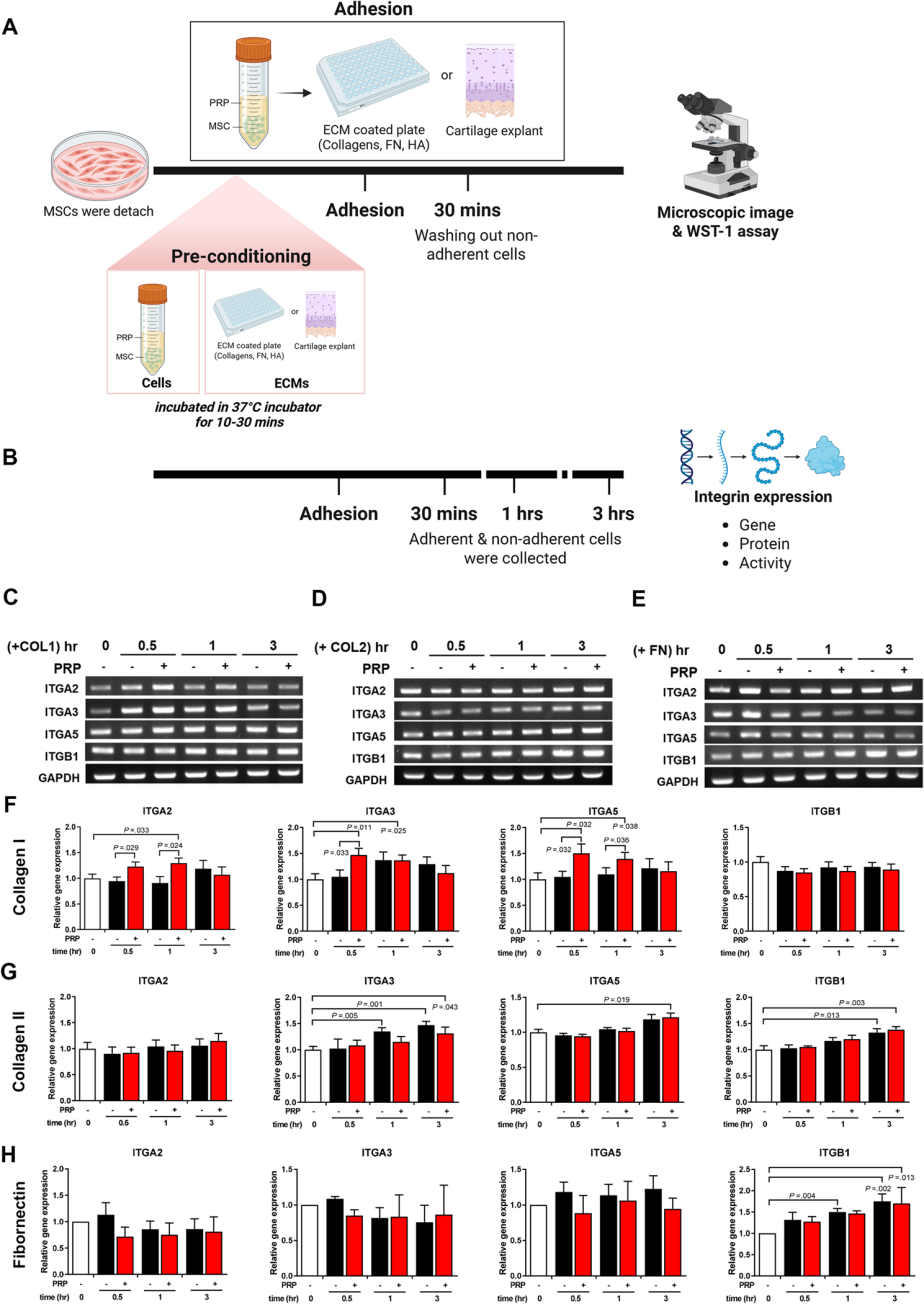
Integrin expression in UC MSCs with or without PRP. UC-MSCs were treated with PRP (without pre-conditioning) and allowed to adhere for 0.5, 1, and 3 h, and integrin gene expression was analyzed using RT-PCR (*n* = 6). (**A**) Schematic representation of cell-ECM adhesion, showing both pre-conditioning and non-pre-conditioning conditions used throughout the study. (**B**) Schematic for analyzing integrin expression at both the gene and protein levels. (**C**, **F**) Integrin expression was analyzed by RT-PCR in type II collagen. (**D**, **G**) Integrin expression was analyzed by RT-PCR in type I collagen. (**E**, **H**) Integrin expression was analyzed by RT-PCR in fibronectin. The mRNA levels of integrin were normalized to GAPDH. All bar charts represent mean ± standard error

To investigate which subunit of integrin mediates the adhesion of MSCs to ECM proteins in presence of PRP, neutralizing antibodies were used to inhibit the function of each subunit of integrin or heterodimer of integrin. The integrin beta 1 subunit primarily inhibited the adhesion of MSCs to collagen I, collagen II, and fibronectin (Fig. 2B). Furthermore, the α2ꞵ1 and α5ꞵ1 subunits also contributed to the inhibition of MSC adhesion to fibronectin by 44.07% and 48.72%, respectively. When only the ꞵ1 subunit was inhibited, adhesion was reduced by 39.04% (Fig. 2C). Integrin α2ꞵ1, α5ꞵ1, and ꞵ1 played a role in MSC adhesion to G1 and G3 cartilage, while integrin α2ꞵ1 (35.10%) and ꞵ1 (39.55%) significantly inhibited MSC adhesion to G2 cartilage (Fig. 2D). Regarding the type I collagen-binding integrins, PRP significantly increased the mRNA levels of α2, α3, and α5 integrin by 1.23-fold, 1.47-fold, and 1.50-fold, respectively, at 30 min (Fig. 3C and F).

### PRP promotes MSCs adhesion via increasing the quantity and activity of integrin ꞵ-1 (ITGB-1)

**Fig. 4:**
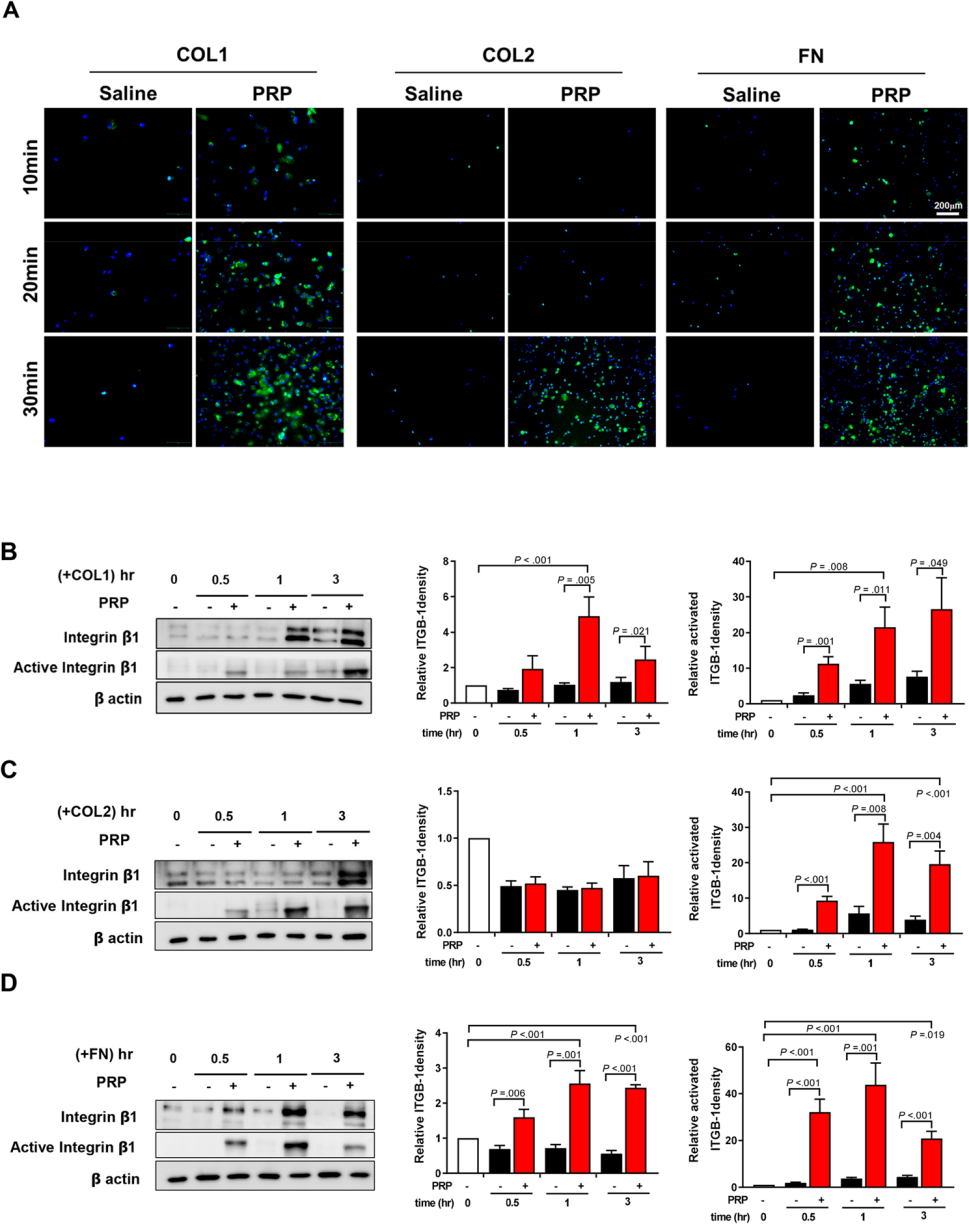
Protein level and activity of integrin beta 1 in UC MSCs. (**A**) Fluorescent images of adherent UC MSCs at 10, 20, and 30 min after seeding into ECM pre-coated plates (*n* = 6). UC MSCs were labeled with DAPI (blue), and activated β1-integrins (ITGB-1) were stained with Alexa Fluor 488 (green). (**B**-**D**) Protein expression and activity levels of β1-integrins were confirmed by western blot (*n* = 3). Both adherent and non-adherent cells were collected at 0.5, 1, and 3 h after seeding. The levels of ITGB-1 and active ITGB-1 is normalized to amounts of beta-actin. All bar charts represent mean ± standard error

To identify the effects of PRP on the protein levels of integrin ꞵ1 and its activity, we performed ICC and western blotting. PRP increased the number of MSCs binding to collagen I, collagen II, or fibronectin, and it also gradually increased the levels of activated integrin beta 1 over time, as shown in Fig. 4A. PRP increased the protein level of ITGB-1 by 4.89-fold or 2.56-fold at 1 h, compared to the control group with the cells binding to collagen I or fibronectin. Furthermore, PRP significantly enhanced the activity of ITGB-1 compared to the control group, with the cells binding to collagen I, collagen II, or fibronectin by 21.44-fold, 25.84-fold, or 43.85-fold at 1 h (Fig. 4B and D). Taken together, these results suggest that PRP increases MSC adhesion to collagens or fibronectin primarily by increasing the quantity and activity of ITGB1.

### Pre-conditioning UC-MSCs with PRP enhanced cell-to-matrix adhesion

**Fig. 5:**
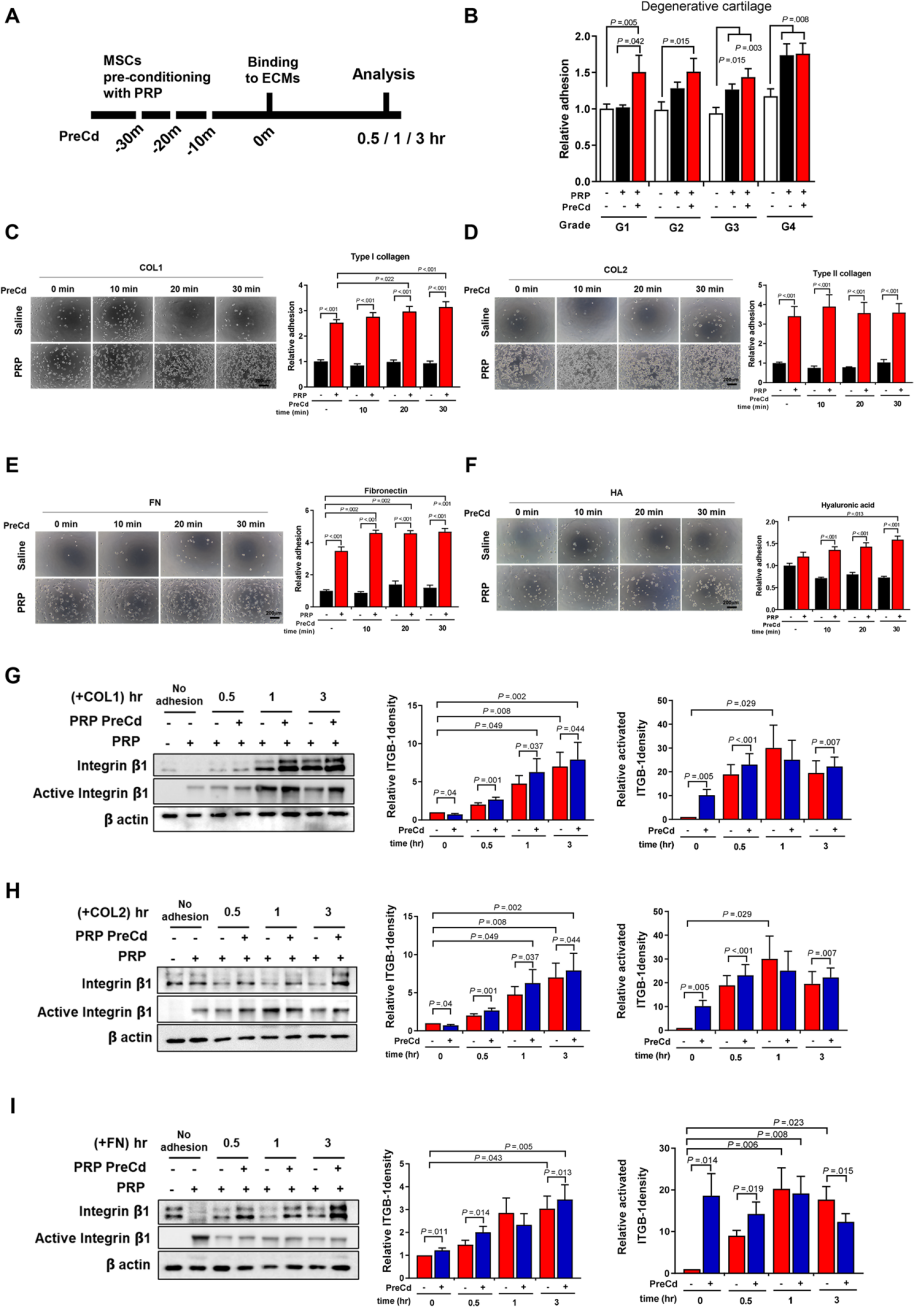
Pre-conditioning (PreCd) UC MSCs with PRP enhanced cell to matrix adhesion. (**A**) A schematic image for cell pre-conditioning. (**B**) After pre-conditioning MSCs with PRP for 30 min, the cells were seeded onto the cartilage explants from G1 to G4 and allowed to adhere for 30 min (*n* = 9). (**C**-**F**) After pre-conditioning UC MSCs with PRP for 30 min, the cells were seeded on (**C**) type I collagen, (**D**) type II collagen, (**E**) fibronectin, and (**F**) hyaluronic acid and allowed to adhere for 30 min. Unattached cells were washed out 30 min after seeding, and adherent cells were measured using a cell viability assay (*n* = 9). (**G**-**I**) Protein expression level and activity level of β1-integrins were confirmed with western blot. Both adherent and non-adherent cells were collected at 0.5, 1, and 3 h after seeding. The level of ITGB-1 and active ITGB-1 is normalized to amount of beta actin. All bar charts represent mean ± standard error

To overcome the limited adhesion of MSCs to ECM, we evaluated the effects of pre-conditioning MSCs with PRP. MSCs were treated under three conditions: (1) without PRP, (2) with PRP, and (3) pre-conditioned with PRP for 30 min before adhesion. The ECM components in cartilage explants vary slightly depending on their degenerative level. Pre-conditioning MSCs with PRP for 30 min further increased the adhesion of MSCs to G1 (1.51-fold) and G2 (1.51-fold) cartilage (Fig. 5B). However, PRP without pre-conditioning had equally effective effects on G3 and G4 cartilage. Therefore, the strategy of pre-conditioning cells with PRP is more effective for early degenerative cartilage defects than for severe cartilage damage. To evaluate the pre-conditioning effects of PRP on MSC adhesion to individual ECMs, the number of adherent cells on type I collagen, type II collagen, fibronectin, and hyaluronic acid was measured (Fig. 5C and F). Pre-treating MSCs with PRP further increased their adhesion to type I collagen, fibronectin, and hyaluronic acid by 1.24-fold, 1.35-fold, and 1.32-fold, respectively, compared to the non-preconditioned PRP group.

### Pre-conditioning MSC with PRP enhances cell-to-cartilage explants adhesion via altered ITGB-1 into active form before its interaction with matrix molecules

**Fig. 6:**
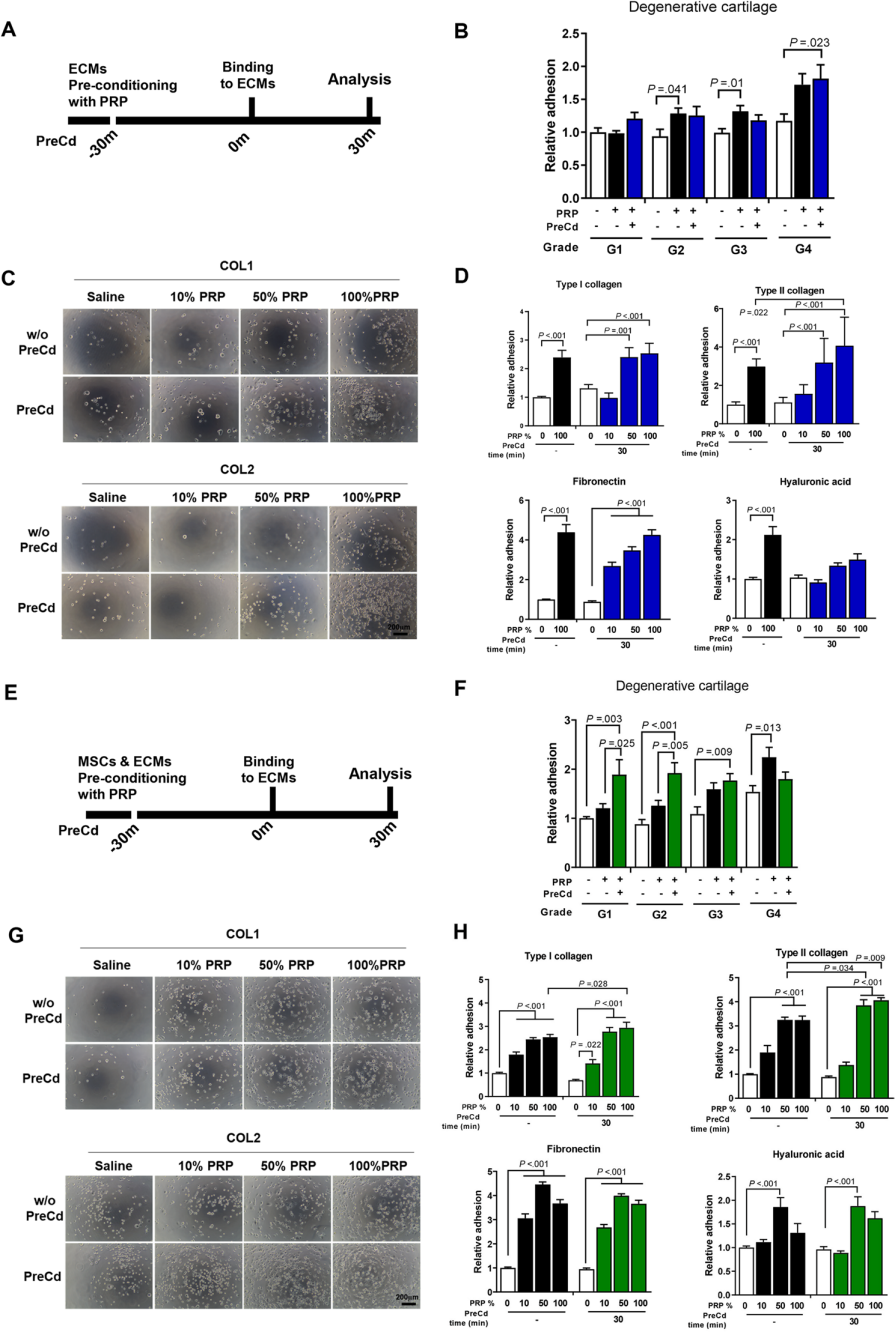
(**A**–**D**) ECM proteins were pre-conditioned for 30 min with 10%, 50%, or 100% PRP, each prepared by dilution in saline, before cell-to-matrix interaction. (**B**) After pre-conditioning the cartilage explants from G1 to G4, adherent cells on cartilage explants were measured after 30 min of adhesion (*n* = 9). (**C**) Representative images showing higher percentages of PRP-conditioned collagens that enhance cell adhesion. (**D**) After pre-conditioning with ECM proteins, adherent cells were measured using a cell viability assay (*n* = 9). (**E**-**H**) Both cells and ECM proteins were pre-conditioned with PRP for 30 min individually, and then conditioned cells were seeded onto conditioned ECM proteins. (**F**) After pre-conditioning both cells and cartilage explants from G1 to G4, adherent cells on cartilage explants were measured after 30 min of adhesion (*n* = 9). (**G**) Representative images showing higher percentages of PRP-conditioned collagens that enhance cell adhesion. (**H**) After pre-conditioning cells and ECM proteins, adherent cells were measured using a cell viability assay (*n* = 9). All bar charts represent mean ± standard error

The protein levels of integrin beta 1 were investigated using a western blot to study the effects of pre-conditioning MSCs with PRP on their adhesion to ECM proteins. The results showed an increase in the protein quantity of integrin beta 1 within 30 min, which further increased over the next three hours during cell-to-matrix interaction with type I collagen or fibronectin (Fig. 5G and I). Interestingly, the pre-treatment of MSCs with PRP alone resulted in the activation of integrin beta 1 in the absence of cell-to-ECM interaction, with levels increasing by 10.172-fold for Col1 (Fig. 6G), 3.014-fold for Col2 (Fig. 6H), and 20.13-fold for FN (Fig. 6I), compared to the control.

### Pre-conditioning ECM proteins with PRP enhances cell-to-matrix adhesion

The effects of PRP and pre-conditioning with PRP on cartilage explants were investigated.

Treating cartilage explants with PRP enhanced cell-to-matrix adhesion in G1, G2, and G3 cartilage by 1.28-fold, 1.32-fold, and 1.72-fold, respectively (Fig. 6B). No significant difference was observed between the pre-conditioning group and the PRP group. The 10%, 50%, and 100% of PRP used for ECM pre-conditioning were chosen to simulate the diluted effects of PRP in the joint space, where it is continuously diluted due to the open, circulating nature of the joint. In terms of matrix conditioning, using a higher concentration of PRP (around 50–100%) is more important for advancing cell adhesion than the duration of conditioning (Fig. 6C and D). However, for type II collagen, pre-conditioning the matrix with PRP is effective, further enhancing its cell adhesion by 1.36-fold compared to the PRP group. The representative images of adherent MSCs on FN and HA are shown in Supplementary Fig. 4.

### Pre-conditioning both UC MSCs and ECM proteins with PRP has synergistic effects on cell-to-matrix adhesion

Pre-conditioning MSCs and cartilage explants with PRP showed enhanced adhesion by 1.89-fold, 1.92-fold, 1.77-fold, and 1.80-fold in G1, G2, G3, and G4, respectively, compared to the control group. The pre-conditioning group significantly enhanced cell-to-cartilage adhesion by 1.57-fold and 1.53-fold in G1 and G2 cartilage, respectively, compared to the PRP group (Fig. 6F). This demonstrated that conditioning with PRP for 30 min is effective on mild degenerative cartilage but offers no further improvement in G3 to G4 cartilage since PRP is already effective in enhancing their adhesion. Pre-conditioning both MSCs and ECM with PRP showed synergistic effects on cell-ECM adhesion (Fig. 6G and H). MSCs significantly increased their adhesion to collagen I, collagen II, fibronectin, and hyaluronic acid by 2.94-fold, 4.06-fold, 3.66-fold, and 1.62-fold, respectively, compared to the control group. PRP with a concentration of 100% resulted in the highest cell adhesion to collagen I and collagen II by 1.16-fold and 1.25-fold, respectively, compared to non-preconditioned PRP groups. The representative images of adherent MSCs on FN and HA are shown in Supplementary Fig. 5.

### PRP enhances cartilage regeneration in the rat osteochondral defect model

**Fig. 7:**
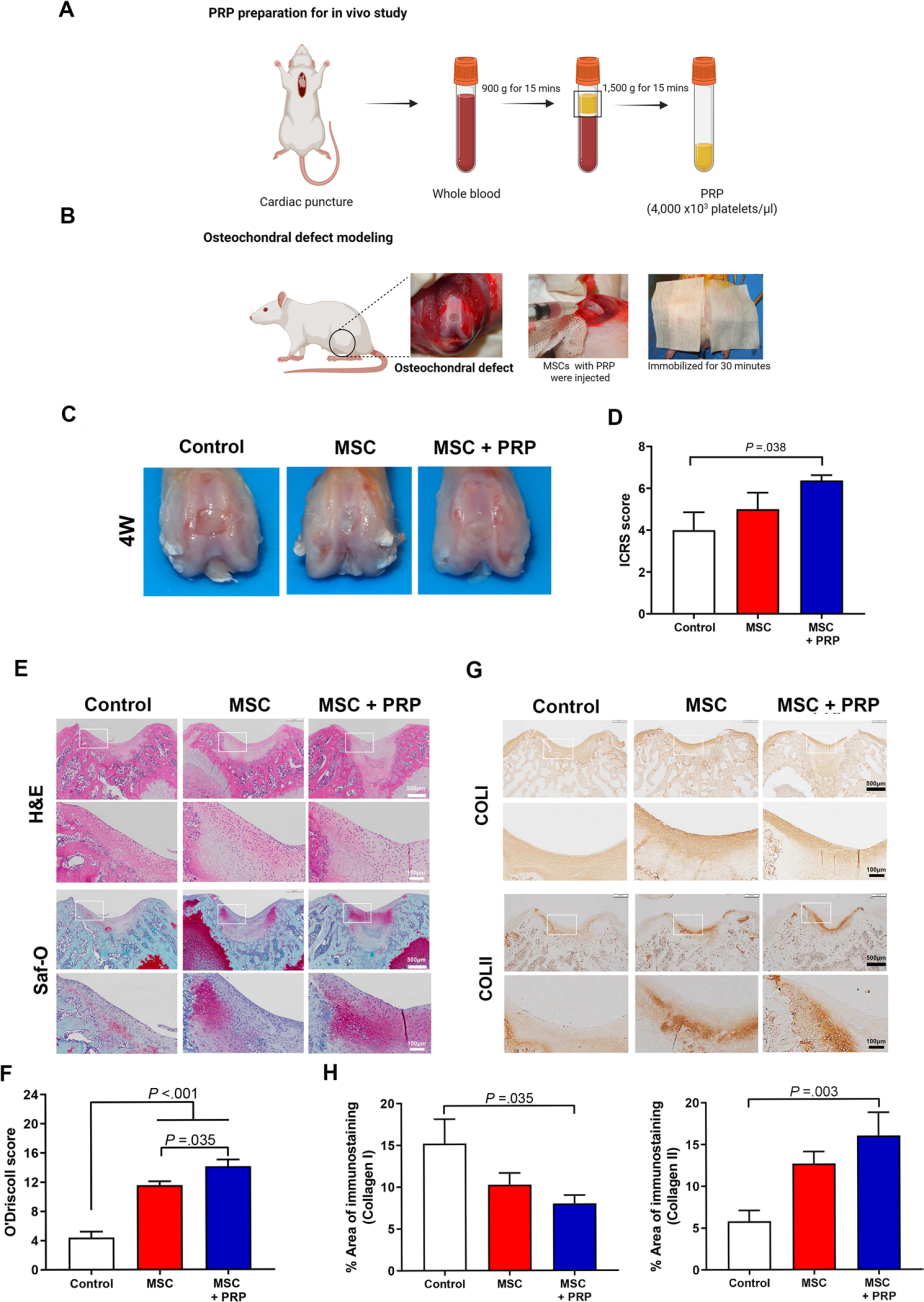
UC MSCs were implanted on the osteochondral defect, followed by 30 min of PRP conditioning to evaluate cartilage regeneration. (**A**-**B**) Schematic representation of PRP preparation for the in vivo study and the surgical procedure. MSCs with or without PRP were implanted into the osteochondral defect and immobilized for 30 min. (**C**) Macroscopic appearance of regenerated cartilage at week 4. (**D**) Cartilage regeneration was evaluated using the ICRS scoring system. (**E**) Hematoxylin and eosin (H&E) and Safranin-O (Saf-O) staining were used for microscopic evaluation. (**F**) Cartilage regeneration was quantified using the O’Driscoll scoring system at week 4. (**G**) IHC staining of type I collagen (COL1) and type II collagen (COLII). (**H**) Quantitation of COL1 and COLII in cartilage using ImageJ. Scale bar, 500 μm. All bar charts represent mean ± standard error

The mean concentrations of platelets, red blood cells, white blood cells, and fibrinogen are shown in supplementary Table 1. The macroscopic evaluation, including the parameters of the degree of defect repair, integration to the border zone, and macroscopic appearance, was measured using the ICRS scoring system (Fig. 7C and D). The UC MSCs + PRP group showed significant cartilage repair in the overall repair score (6.38 ± 0.62) compared to the control group (4.00 ± 0.86; *P* = .038). In the histological evaluation, the UC MSCs (11.56 ± 0.53) and UC MSCs + PRP (14.13 ± 0.89) groups showed significantly regenerated cartilage defects compared to the control group (4.40 ± 0.82; both *P* < .001) at week 4 (Fig. 7E and F; see supplementary Table 5 for histological evaluation). PRP further enhanced cartilage regeneration compared to the MSC group (*P* = .035) in the overall assessment score. This enhancement was attributed to PRP improving the bonding, cellularity, and integration of regenerated cartilage to adjacent cartilage (freedom from degenerate changes; *P* = .017). In our osteochondral models, fibrocartilage was formed in the control group at week 4. In our osteochondral models at week 4, fibrocartilage was formed in the control group, which lacked GAG content and widely expressed type I collagen (Fig. 7G, H). In contrast, the repaired cartilage with MSCs showed a 32.42% decrease in type I collagen expression and a 218.6% increase in type II collagen expression compared to the control group. Compared to the control group, treatment of MSCs with PRP further decreased type I collagen expression by 47.35% and increased type II collagen expression by 275.9%. Thus, MSCs with PRP significantly reduced type I collagen and enhanced type II collagen in the repaired area at week 4.

## Discussion

In this study, we applied PRP to MSCs and degenerative cartilage to enhance cell interaction with ECMs. The most important findings of this study are as follows; (1) PRP significantly increased the adhesion of MSCs to collagens, fibronectin, and degenerative osteochondral explants from G1 to G3. (2) ITGB1 plays a central role in UC MSC adhesion, and inhibiting ITGB1 decreased the adhesion of MSCs to COLI, COLII, and FN by 46.33%, 57.10%, and 40.86%, respectively. (3) Pre-conditioning of MSCs with PRP altered ITGB1 into an active form before cell-to-ECM interaction. (4) Pre-conditioning cells and degenerative osteochondral explants with PRP had synergistic effects on cell-to-ECM interaction in G1 to G3 explants compared to the non-conditioning PRP group. 4) In histological evaluation, the O’Driscoll scores of UC MSCs (11.56 ± 0.53, *p* < .001) and UC MSCs + PRP group (14.13 ± 0.53, *p* < .001) were significantly higher than the control group (4.40 ± 0.82). PRP as a vehicle of MSCs showed a higher O’Driscoll score than MSCs with saline by 1.2-fold (*p* = .035). To the best of our knowledge, this is the first study demonstrating PRP is a possible vehicle for MSC delivery of their ability to increase the quantity and activity of ITGB1 to facilitate the adhesion of MSCs to cartilage ECM. Moreover, increasing the initial MSC engraftment on degenerative cartilage with PRP improved the regenerative effects of UC MSCs on osteochondral defects.

Synovial fluid (SF) is a pooled microenvironment that reflects the severity of disease progression and serves as a site where mesenchymal stem cells (MSCs) are first encountered in cell therapy. Cell adhesion is reported to be inhibited by macromolecules, including aggrecan, decorin, and biglycan. Removal of proteoglycans from cartilage surfaces may expose underlying collagen, which helps cell-to-matrix adhesion [23, 44]. Hyaluronic acid aids in lubrication, shock absorption, and chondroprotection. However, osteoarthritic SF and high molecular weight HA inhibited MSC adhesion, and MSC adhesion to cartilage was rescued by hyaluronidase treatment [45]. In patients undergoing osteoarthritis, synovitis develops, inducing effusion of inflamed SF rich in inflammatory factors such as IL-1, IL-6, IL-8, IL-17, and TNF-α. Fan et al. reported that the inflamed SF microenvironment affects the internal living conditions of MSCs and could further worsen the joint microenvironment when using MSCs [46]. Our findings are in line with previous reports that OA SF inhibited MSC adhesion to collagen and that removing the SF to lower levels could rescue cell adhesion to collagen. In clinical practice, washing out inflamed SF and using adjuvants that aid cell adhesion and alleviate the hostile microenvironment before cell therapy may be suggested, as OA SF inhibits cell adhesion to the matrix and may worsen the efficacy of MSC therapy.

To enhance the therapeutic effect of MSCs on cartilage regeneration, stem cell engraftment at the injury site is important [8, 21]. Since cartilage surface adhesion is inhibited by macromolecules, degenerative cartilage where collagens are exposed is more conducive to cell adhesion [23, 44]. Our study also showed that MSC adhesion to grade 4 (bone-exposed) cartilage is significantly higher than its adhesion to grade 1 by 1.93-fold. Therefore, to have therapeutic effects in the early to mid-stages of OA, it is important to overcome the difficulties in cell-to-matrix interaction, especially from grade 1 to grade 3. Recent studies have made efforts to enhance cell adhesion. Magnesium, a co-factor of integrin, was reported to enhance the adhesion of human synovial MSCs to collagen, and osteochondral defects [27]. The use of magnetic labeling of bone marrow MSCs and an external magnet has been proposed to deliver injected cells to defects in mini-pig knees and in five cases of patients [47, 48]. Zwolanek et al. reported an addition of serum increased the MSC-covered area on both the intact and the cartilage defect in a dose-dependent manner [14]. Modifying cell membranes with platelet-derived microparticles promoted the homing of synovial MSCs to cartilage in rats via acquiring platelet-specific adhesion molecules, which resulted in facilitating cartilage regeneration [8]. In our results, improving the adherent ability of MSCs to degenerative cartilages from grade 2 to grade 4 without a time-consuming modification was shown by simply treating cells with allogeneic PRP.

Integrins are important membrane-bound proteins that mediate cell adhesion to major ECM proteins present on degenerative cartilage surfaces. Since MSCs derived from various tissues, donor age, culture conditions, and isolation reflect heterogeneity, different expression levels of integrin subunits were detected [26, 49]. Integrin α3 and ꞵ1 subunits are involved mainly in the synovial MSC adhesion to collagen [27, 28]. Wherein, the adhesion of bone marrow MSCs to type I collagen was mainly interacted through integrin α2ꞵ1 [50]. Nonetheless, ITGB1 is known to play the most critical role in MSCs binding to multiple ECM proteins, including fibronectin, laminin, and collagen [25]. Our study confirmed that inhibition of ITGB1 decreased the adhesion of MSCs to COLI, COLII, and FN by 46.33%, 57.10%, and 40.86%, respectively, highlighting the central role of ITGB1 in UC MSCs to ECM binding. Interestingly, PRP did not increase the mRNA expression level of ITGB1, but significantly increased the protein expression level and their activity. The cell binding to the target ECM happens at the very beginning after the injection, therefore the recycling of pre-existing active and inactive ITGB1 could be more critical than modulating the mRNA level of integrins. Therefore, modulating integrin conformation to its active form using PRP enhances the adhesion of MSCs to the ECM proteins that constitute cartilage.

Pre-conditioning MSCs to enhance their adhesion to the target ECM is important in that it not only increases the number of efficacious MSCs for stem cell therapy but also reduces apoptotic cell death caused by insufficient matrix support [13]. Warstat et al. reported that TGF-ꞵ enhances the integrin α2ꞵ1-mediated adhesion of MSCs to type I collagen by up-regulating the expression of integrin subunits mediated by Smad2 [50]. Interaction of integrin α2ꞵ1 and α11ꞵ1 to collagen regulated the survival of MSCs and integrin-dependent of mammary and intestinal epithelial cells to the ECM avert anoikis through the activation of protein kinases B survival pathway and the prevention of BAX [51]. Platelet-rich clot releasate on rat bone marrow MSCs reduced apoptosis and promoted regenerative function in a hostile microenvironment via paracrine or autocrine factors [52]. Stemness properties of adipose tissue-derived MSCs were enhanced with activated PRP scaffold, and enhanced cell proliferation and differentiation were identified in the study of Tobita [53]. A recent study of Fukui et al. found that PRP aids the proliferation, migration, and adhesion to type I collagen of human adipose-derived stem/stromal cells [54]. Our results build on this knowledge by demonstrating that PRP treatment increases ITGB1 activity in MSCs. Notably, we found that pre-conditioning MSCs with PRP converted inactive ITGB1 into its active form even in the absence of cell-to-ECM interaction. It is known that integrin adhesion frequently synergizes with growth factor-dependent cascades [55]. Furthermore, integrins are physically and functionally associated with growth factor receptors, thereby regulating their downstream signaling. The combination of ‘outside-in’ and ‘in-outside’ signaling in integrin leads to intracellular signals that control cell polarity, proliferation, and survival [56]. PRP, a great reservoir for growth factors, which affects cell growth factor receptors. Therefore, the most plausible explanation from our findings is that PRP treatment on cells increases cell adhesion to degenerative cartilage due to growth factors in PRP, which aid the synergistic ‘inside-out’ signaling and ‘outside-in’ signaling of integrin.

Pre-conditioning the hostile disease microenvironment to alleviate host factors affecting the therapeutic outcomes of MSCs before cell therapy can be a strategy [29, 46]. Variations in the host cytotoxic response, inflammation status, and tissue microenvironments such as hypoxia and ECM are important factors in the efficacy of MSCs after administration [57]. In our study, the duration of conditioning ECM proteins was not critical for cell-to-matrix interaction, but the high concentration of PRP was more effective in enhancing their adhesion. PRP concentrations ranging from 50 to 100% effectively increase cell-to-matrix adhesion. One possible reason for this effect is that the soluble factors abundant in PRP, such as growth factors, could non-covalently interact with the endogenous ECM, which may affect the retention time of the crosstalk between integrins and growth factor receptors [58–63]. The activity of cell integrins can also be enhanced by growth factors such as PDGF, bFGF, TGF-β, EGF, IGF, and VEGF [50, 64]. The possible explanation is that growth factors that non-covalently interact with endogenous ECM may affect the retention time of the crosstalk between integrins and growth factor receptors. A second possible reason is that MMPs in PRP could degrade or remodel the ECM, altering its structure and composition [65, 66]. Exposing specific ECM binding sites, such as integrin binding sites, growth factor binding sites, and the RGD sequence, may play a critical role in cell-ECM interactions [67, 68]. Interestingly, pre-conditioning both MSCs and ECM with PRP synergistically enhances their interaction in degenerative cartilage from G1 to G2 compared to the PRP group alone. The adherent effects of PRP were more effective on G3-G4 cartilage than on G1-G2, resulting in no further synergistic effects from using the pre-conditioning technique in G3-G4. This suggests that enhancing cell-ECM interactions in advanced OA is more achievable, but more challenging in early OA. Therefore, to enhance cell-ECM interactions in the early stages of OA, conditioning both cells and ECM with PRP could be a possible strategy.

Histological evaluation showed that both groups (1) UC MSCs alone, (2) UC MSCs + PRP significantly regenerated cartilage defects compared to the control group. Interestingly, the repaired cartilage treated with MSC + PRP showed better cartilage thickness (1.48-fold) and better integration to adjacent cartilage tissue with higher GAG contents and the number of chondrocytes than the group of UC MSCs alone (3.12-fold; *P* = .017). Many studies showed that MSCs without any adjuvant therapies improve pain and function of osteoarthritis [6, 22, 69, 70]. However, clinical trials and preclinical studies have not shown consistent results regarding the healing of damaged tissue after MSC treatment due to variables such as cell culture conditions, pre-conditioning of cells, cell density, and delivery methods [57]. For these factors that influence the survival and engraft rate of MSCs to the damaged tissue, modification of MSCs using several supplements was studied such as collagen gel, fibrin glue, hyaluronic acid, or PRP [71]. To the current knowledge, studies conducted on MSC concurrent use of PRP in terms of cartilage defect are limited to autologous bone marrow or adipose-derived stromal cells (without culture) [72–74]. Koh et al. compared the cartilage repair using 3.0 mL of PRP to infrapatellar fat pad-derived mesenchymal stromal cells (MSC) with PRP. Both PRP and MSC + PRP groups improved the mean Lysholm, Tegner activity scale, and VAS scores to 16 months with no significant difference between the two groups. Nevertheless, the use of expanded MSCs is more promising than the use of autologous MSCs in clinical applications in that the characteristics and the safety profile of the cells are verified before treatment [17]. In addition, it is important to expand cells in large quantities without cell aging and to develop in off-the-shelf use. Therefore, we investigated the ability of cartilage repair using UC MSC, since UC MSCs retain higher proliferative ability without senescence up to passage 20 compared to other adult MSCs [36]. Our study showed that the UC MSCs prevent the forming of fibrocartilage by increasing GAG content and promoting the distribution of type II collagen in repaired cartilage, wherein the expression of type I collagen was markedly downregulated. Several studies showed that initial cell engraftment to the cartilage defect is critical for their therapeutic effects on tissue repair. The combined administration of 5mM magnesium with synovial MSCs to an osteochondral defect not only increased the adhesion of MSCs to the defect and increased the type II collagen expression in the repaired tissue in week 4 compared to the MSC only group in a rabbit model [27]. Liang et al. revealed that decoration of synovial MSC membrane with platelet-derived microparticle enhanced cell adhesion to cartilage and the subchondral bone surface via GPIIb/IIIa, CXCR4, ITGβ1 and ITGα2 [8]. Intra-articular injection of MSCs produced only a thin layer of regenerative tissue, while platelet-derived microparticle-treated MSCs generated a significantly large volume of repaired tissue but failed to produce hyaline cartilage in both groups. These studies showed that initial cell engraftment to the cartilage defect is critical for their therapeutic effects on tissue repair. However, fully elucidating the contribution of injected MSC to cartilage repair is difficult due to the lack of MSC-specific markers *in vivo* [75]. The limitation of our study was that, despite PRP alone still having conflicting results in some studies, its effect on cartilage repair was not investigated in the current study. Several studies have reported significant improvements in pain and function, while others have found no significant differences compared to the placebo treatment [32, 76–78]. To date, current treatments are primarily focused on pain management and function of knee cartilage, and regenerating cartilage defects to hyaline cartilage is still challenging. Although it is limited to animal testing, our study suggests the possibility that PRP is effective in cartilage regeneration by increasing the MSC engraftment to the target.

The current study has some limitations: (1) The factors that affect cell-to-matrix interaction in PRP were not elucidated, leaving a gap in understanding how PRP supports MSC adhesion and engraftment; (2) The initial cell engraftment rate in a rat cartilage defect model was not observed, therefore, the number of cells that adhere, their survival, and the duration of their distribution were not discussed; (3) The mode of action of target pre-conditioning of PRP on cartilage ECMs was not demonstrated, although two possible mechanisms were suggested; (4) The PRP without MSC group was not investigated in the animal study, so the independent effects of PRP alone on cartilage repair and regeneration were not determined.

## Conclusions

In summary, pre-conditioning MSCs and cartilage ECM with PRP synergistically enhanced MSC adhesion to the cartilage ECM by increasing both the quantity and activation of ITGB1 in MSCs, thereby enhancing the therapeutic effects of MSCs on articular cartilage regeneration in a rat osteochondral defect model. Allogeneic PRP could be a treatment option to enhance the initial engraftment efficacy of MSC therapy.

## Supplementary Information

Below is the link to the electronic supplementary material.

Supplementary Material 1

## Data Availability

The datasets used and/or analyzed during the current study are available from the corresponding author on reasonable request.
