# Supplementary material for: Platelet-rich plasma enhances local homing of umbilical cord-derived mesenchymal stem cells to articular cartilage by increasing the quantity and activation of integrin ꞵ1
Source: Stem Cell Res Ther. 2025 Sep 2;16:487. doi: 10.1186/s13287-025-04593-y (PMC12403387; doi:10.1186/s13287-025-04593-y)
Supplement: Supplementary file 1 — Supplementary Material 1 [file 13287_2025_4593_MOESM1_ESM.docx]

Platelet-rich Plasma Enhances Local Homing of Umbilical Cord-Derived Mesenchymal Stem Cells to Articular Cartilage by Increasing the Quantity and Activation of Integrin ꞵ1

Supplementary table 1. Characteristics of rat PRPs used for *In vivo*

| Characteristics of rat PRPs used for *In vivo* | | | | |
| --- | --- | --- | --- | --- |
| Counts of platelets, RBCs, and WBCs; concentration of fibrinogen | | | |  |
|  | Platelets, | RBCs, | WBCs, | Fibrinogen, |
|  | ×10^3^/μL | ×10^6^/μL | ×10^3^/μL | mg/dL |
| Whole blood | 769.00 ± 2.83 | 6.88 ± 0.18 | 4.05 ± 1.15 | 136.15 ± 42.50 |
| PPP | 33.50 ± 9.19 | 0.02 ± 0.01 | 0.01 ± 0.00 | 212.65 ± 16.33 |
| PRP | 4337.50 ± 35.36 | 0.41 ± 0.004 | 11.45 ± 1.26 | 242.19 ± 153.35 |
| Characteristics of rat PRPs used for *In vivo*. The mean concentrations of platelets, red blood cells, white blood cells, and fibrinogen. Data are presented as mean ± SD. PPP, platelet-poor plasma; PRP, platelet-rich plasma; RBC, red blood cell; WBC, white blood cell. | | | | |

Supplementary table 2. List of antibodies

| Antibodies |  |  |  |
| --- | --- | --- | --- |
| Target protein | Catalogue number, Company / Application | Host species | Working dilution |
| IgG1 Isotype Control | 5415, Cell Signaling | anti-mouse | 10 µg/mL |
|  | Neutralizing |  |  |
|  |  |  |  |
| Integrin α2 | sc-53502, Santacruz | anti-mouse | 10 µg/mL |
|  | Neutralizing |  |  |
|  |  |  |  |
| Integrin α3 | sc-13545, Santacruz | anti-mouse | 10 µg/mL |
|  | Neutralizing |  |  |
|  |  |  |  |
| Integrin α5 | MAB1864, R&D systems | anti-mouse | 10 µg/mL |
|  | Neutralizing |  |  |
|  |  |  |  |
| Integrin β1 | MAB17781, R&D systems | anti-mouse | 10 µg/mL |
|  | Neutralizing |  |  |
|  |  |  |  |
| Integrin β1 | ATGA0485, NKMAX BIO | anti-mouse | 1:500 |
|  | Western blot |  |  |
|  |  |  |  |
| Activated integrin β1 | MAB2079Z, Sigma-Aldrich | anti-mouse | 1:1000 |
|  | Western blot, Immunocytochemistry |  |  |
|  |  |  |  |
| β-actin | sc47778, Sactacruz Bio | anti-mouse | 1:1000 |
|  | Western blot |  |  |
|  |  |  |  |
| HRP-conjugated | SA001, Gendpot | anti-mouse | 1:4000 |
|  | Western blot |  |  |
|  |  |  |  |
| Alexa Fluor 488 | A-21121, ThermoFisher | anti-mouse | 1:200 |
|  | Immunocytochemistry |  |  |

| Supplementary table 3. Modified classification of Yulish and Outerbridge  **Modified classification of Yulish and Outerbridge** | | | | |
| --- | --- | --- | --- | --- |
| Yulish | |  | Outerbridge | |
| Grade | Description |  | Grade | Description |
| 0 | Normal |  | 0 | Normal |
| 1 | Normal contour ± abnormal signal |  | 1 | Softening, without morphologic defect |
| 2 | Partial-thickness defect less than 50% of the cartilage thickness |  | 2 | Partial-thickness defect less than 50% of the cartilage thickness |
| 3 | Partial-thickness defect from 50% to 100% of the cartilage thickness |  | 3 | Partial-thickness defect from 50% to 100% of the cartilage thickness |
| 4 | Full-thickness cartilage loss |  | 4 | Complete loss with exposure of subchondral bone |

Supplementary table 4. Primer nucleotide sequences

| Primer nucleotide sequences | |  |
| --- | --- | --- |
| Target gene | Primer sequence | Product length (bp) |
| ITGA2 | F: 5' GGCACTATCCGCACAAAGTA 3' | 497 |
|  | R: 5' TCCACACGCAAATCCAAAGA 3' |  |
|  |  |  |
| ITGA3 | F: 5' CCTCACTCCTTCTTCATGGC 3' | 348 |
|  | R: 5' AGGTCTTGTGGACGATGTTG 3' |  |
|  |  |  |
| ITGA5 | F: 5' CCAGCAGGGAGTAGTGTTTG 3' | 341 |
|  | R: 5' TTCCAGAAGCATTGAGGCAG 3' |  |
|  |  |  |
| ITGB1 | F: 5' GTGCATGTGACTGTTCTTTGG 3' | 304 |
|  | R: 5' GACACAGGATCAGGTTGGAC 3' |  |
|  |  |  |
| GAPDH | F: 5' AAATCCCATCACCATCTTCCAG 3' | 313 |
|  | R: 5' CATGAGTCCTTCCACGATACC 3' |  |

Supplementary table 5. Histological Evaluation of cartilage repair

| **Histological Evaluation using O'Driscoll scoring system** | | | | | | | | | | |  |
| --- | --- | --- | --- | --- | --- | --- | --- | --- | --- | --- | --- |
| **Parameter** | | **Control** |  | **MSC** | |  | **MSC + PRP** | | | | |
|  |  | Mean ± SE |  | Mean ± SE | P Value^a^ |  | Mean ± SE | P Value^a^ | | P Value^b^ | |
| Nature of predominant tissue | Cellular morphology | 0.60 ± 0.31 |  | 2.22 ± 0.15 | <0.001 |  | 2.67 ± 0.25 | <0.001 | | 0.427 | |
|  | Saf-O staining | 0.80 ± 0.33 |  | 2.33 ± 0.29 | 0.003 |  | 2.53 ± 0.24 | 0.001 | | 1.000 | |
| Strucural characteristics | Surface regularity | 1.10 ± 0.10 |  | 1.44 ± 0.17 | 0.482 |  | 1.60 ± 0.16 | 0.156 | | 1.000 | |
|  | Structural integrity | 0.20 ± 0.13 |  | 1.00 ± 0.00 | <0.001 |  | 1.13 ± 0.09 | <0.001 | | 0.582 | |
|  | Thickness | 0.20 ± 0.13 |  | 0.94 ± 0.13 | 0.009 |  | 1.40 ± 0.19 | <0.001 | | 0.103 | |
|  | Bonding to the adjacent cartilage | 0.10 ± 0.10 |  | 1.00 ± 0.14 | 0.001 |  | 1.40 ± 0.16 | <0.001 | | 0.142 | |
| Freedom from cellular changes or degeneration | Hypocellularity | 0.20 ± 0.13 |  | 1.28 ± 0.16 | <0.001 |  | 1.80 ± 0.17 | <0.001 | | 0.065 | |
|  | Chondrocyte clustering | 1.20 ± 0.20 |  | 1.06 ± 0.21 | 1.000 |  | 0.73 ± 0.18 | 0.431 | | 0.708 | |
| Freedom from degenerative changes in adjacent cartilage | | 0.00 ± 0.00 |  | 0.28 ± 0.11 | 0.690 |  | 0.87 ± 0.22 | 0.002 | | 0.017 | |
| Overall repair score | | 4.40 ± 0.82 |  | 11.56 ± 0.53 | <0.001 |  | 14.13 ± 0.89 | <0.001 | | 0.035 | |
| Data were analyzed with one-way analysis of variance (ANOVA) with post hoc analysis using Bonferroni multiple comparison test. | | | | | | | | |  | |  |
| ^a^P value compared to the control group | |  |  |  |  |  |  |  | |  | |
| ^b^P value compared to the MSC group | |  |  |  |  |  |  |  | |  | |

Supplementary figure 1. Gel images


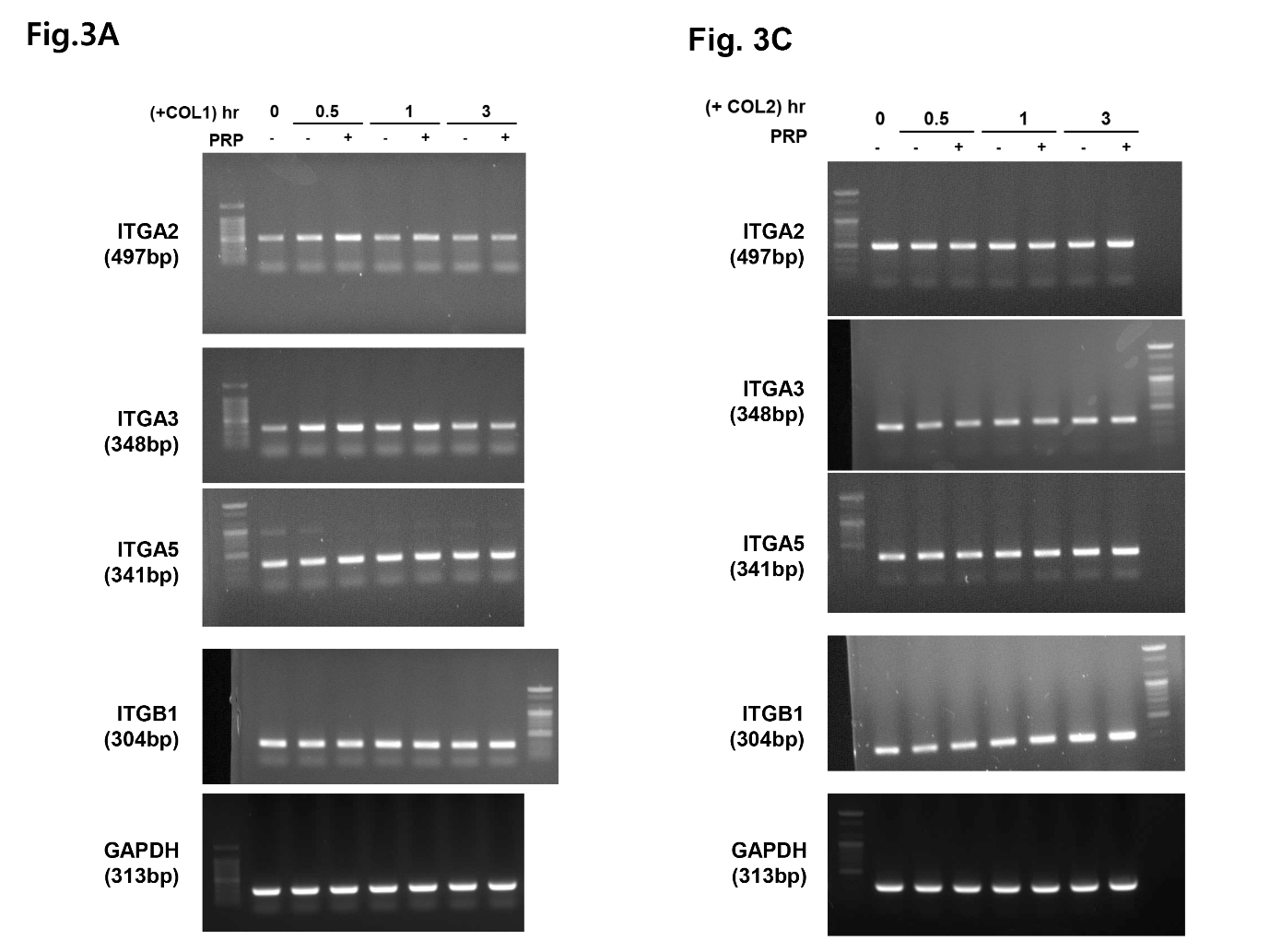


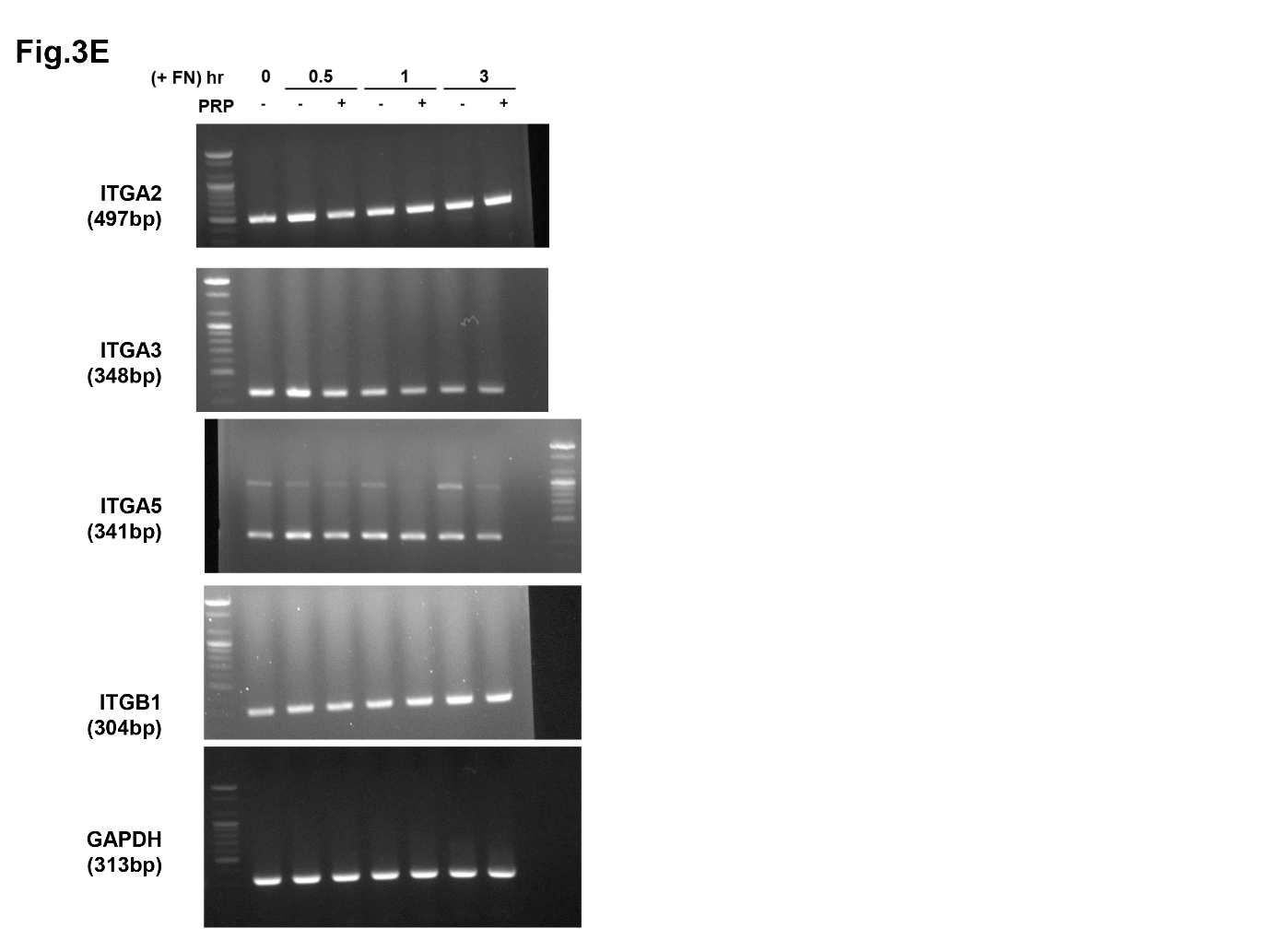


Supplementary figure 2. Blot images for PRP treatment without conditioning


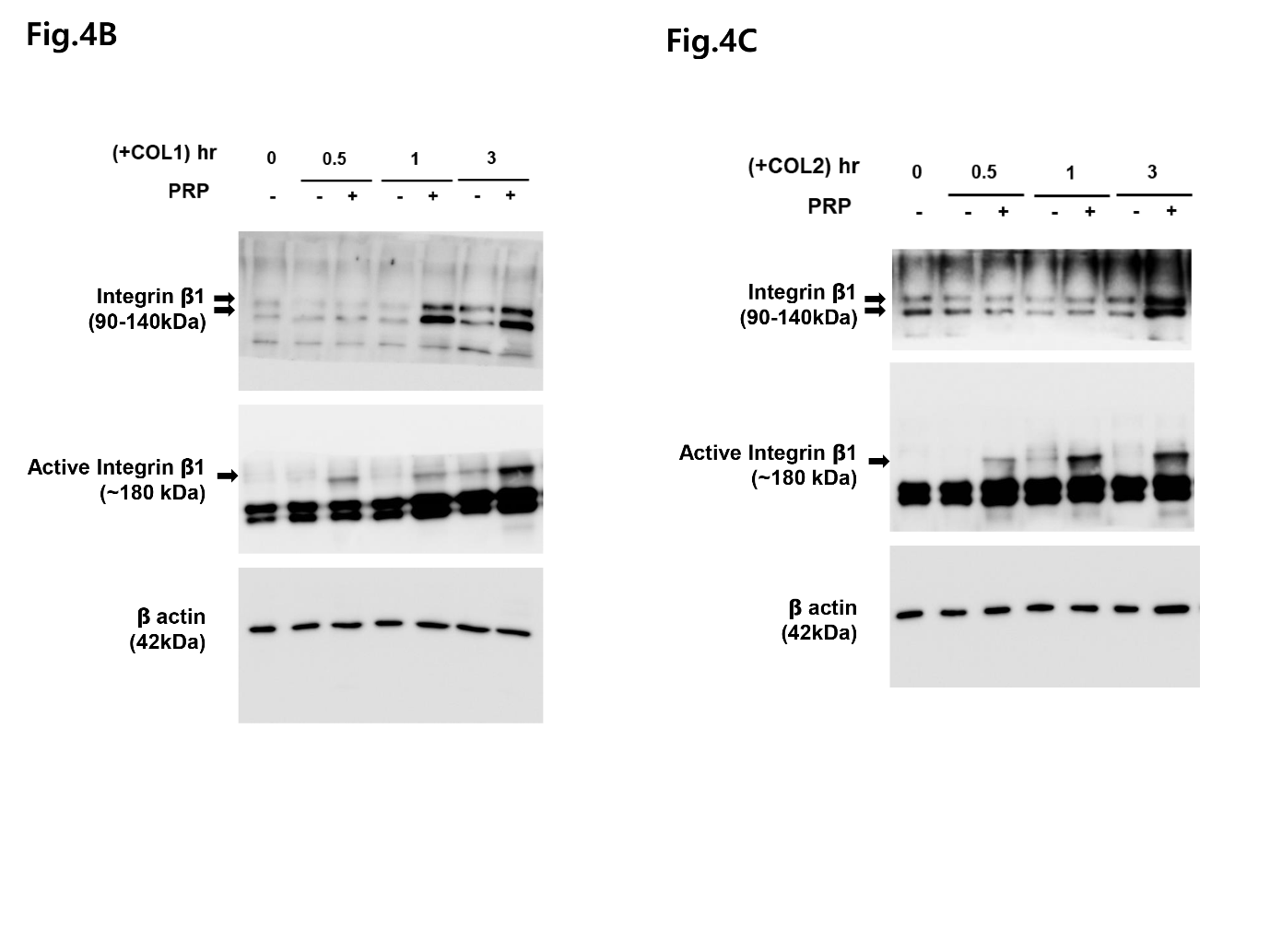


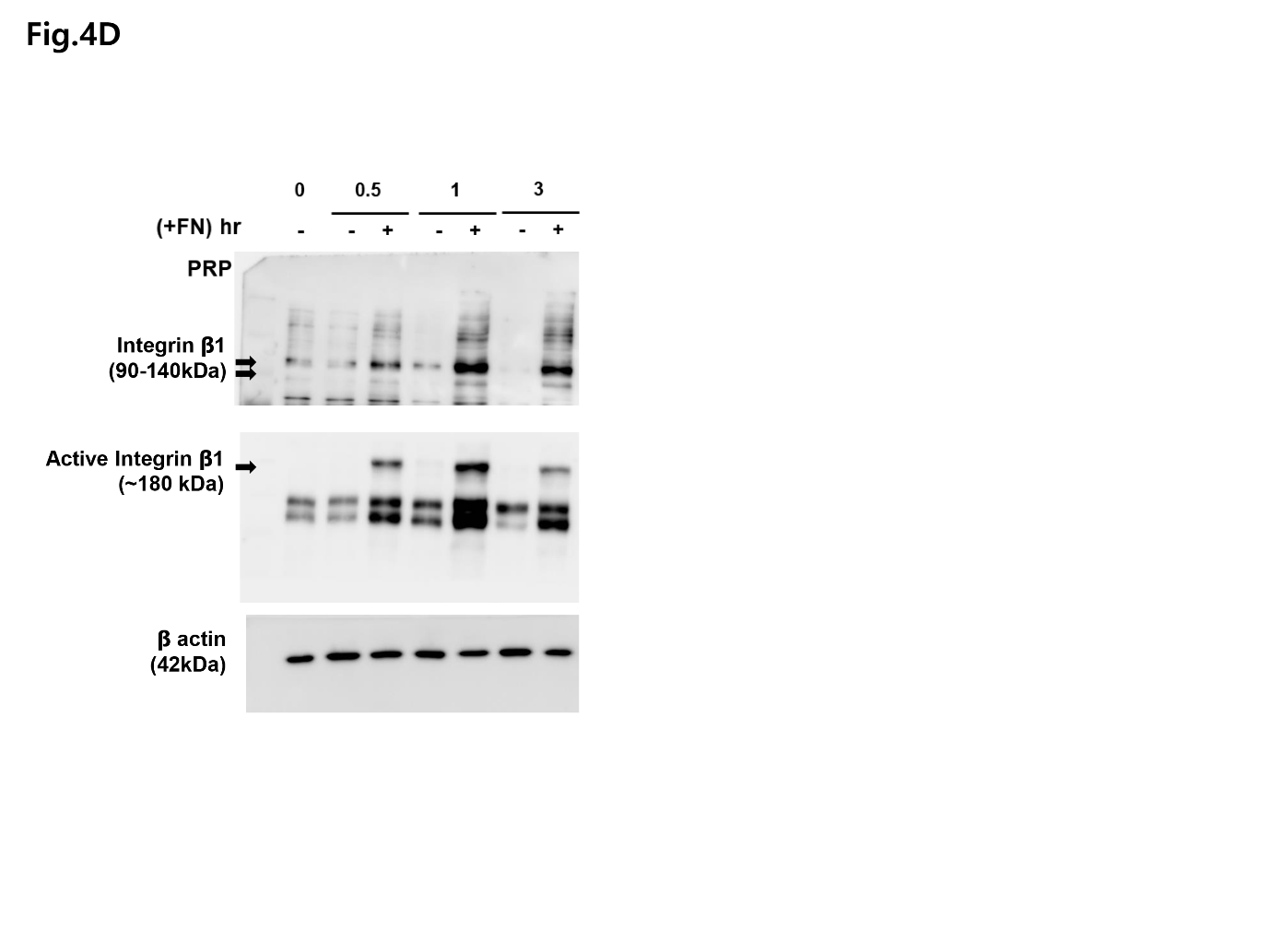


Supplementary figure 3. Blot images for pre-conditioning MSCs with PRP


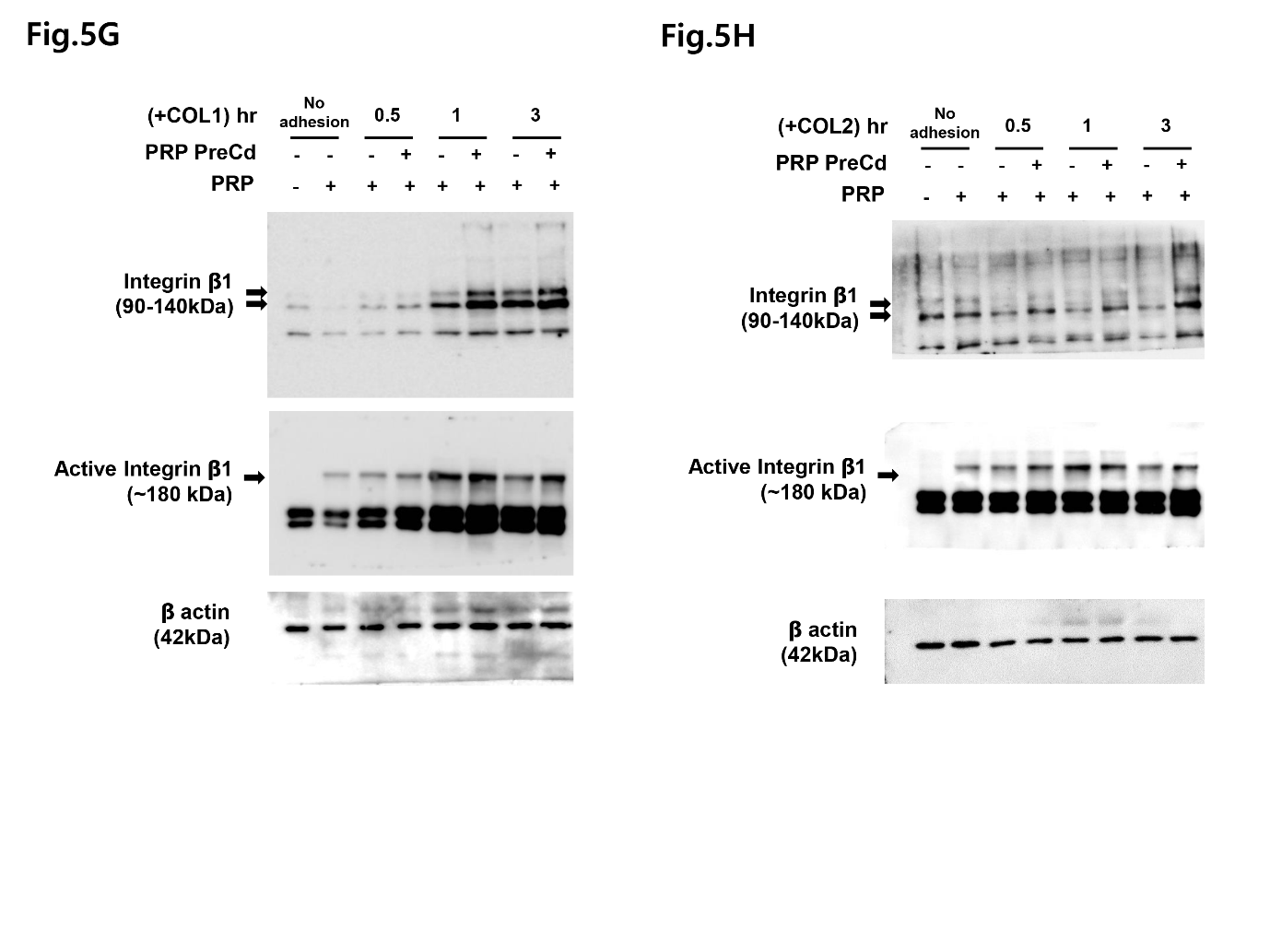


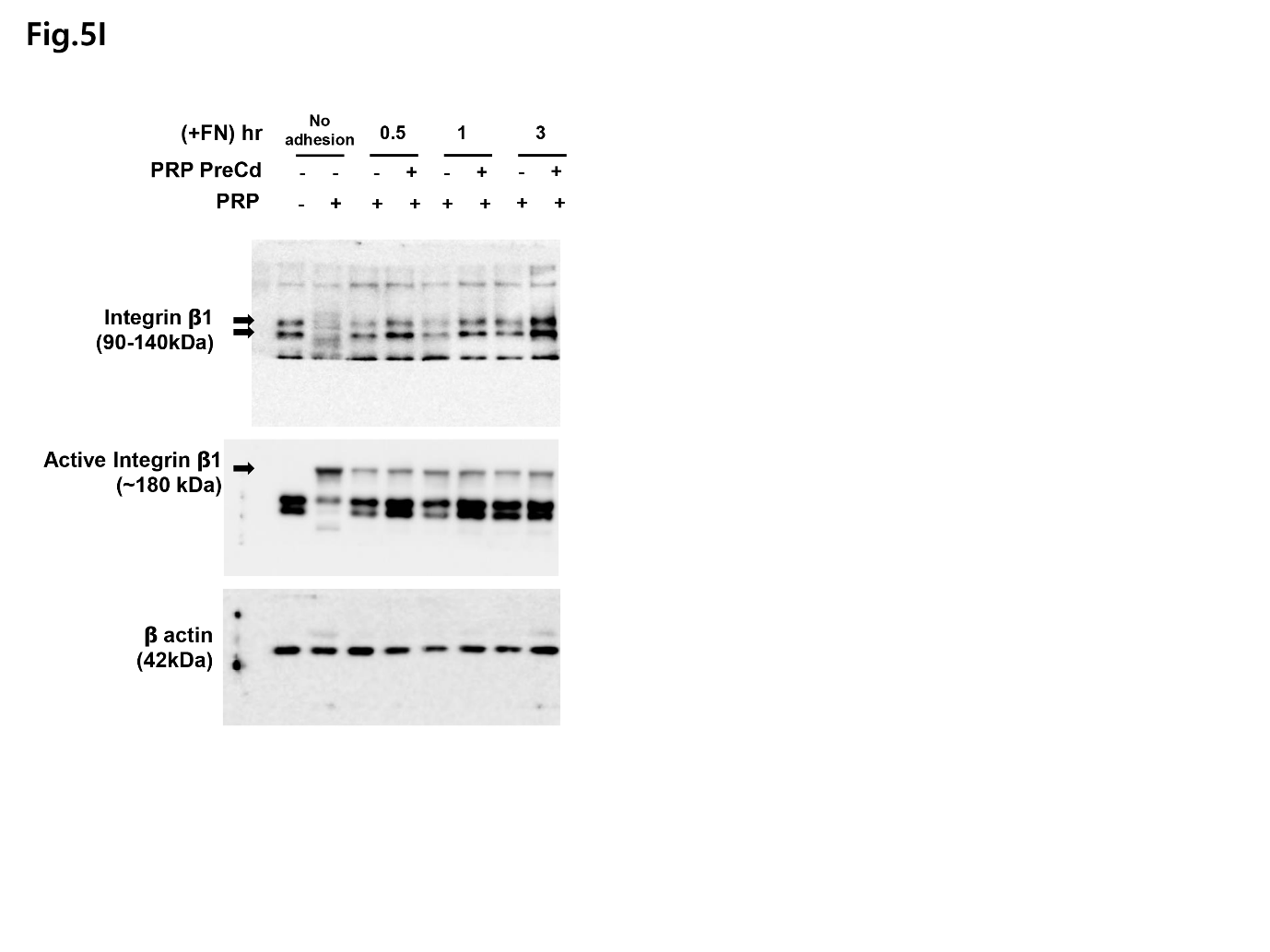


Supplementary figure 4. Representative images of adherent MSCs on FN and HA following ECM preconditioning


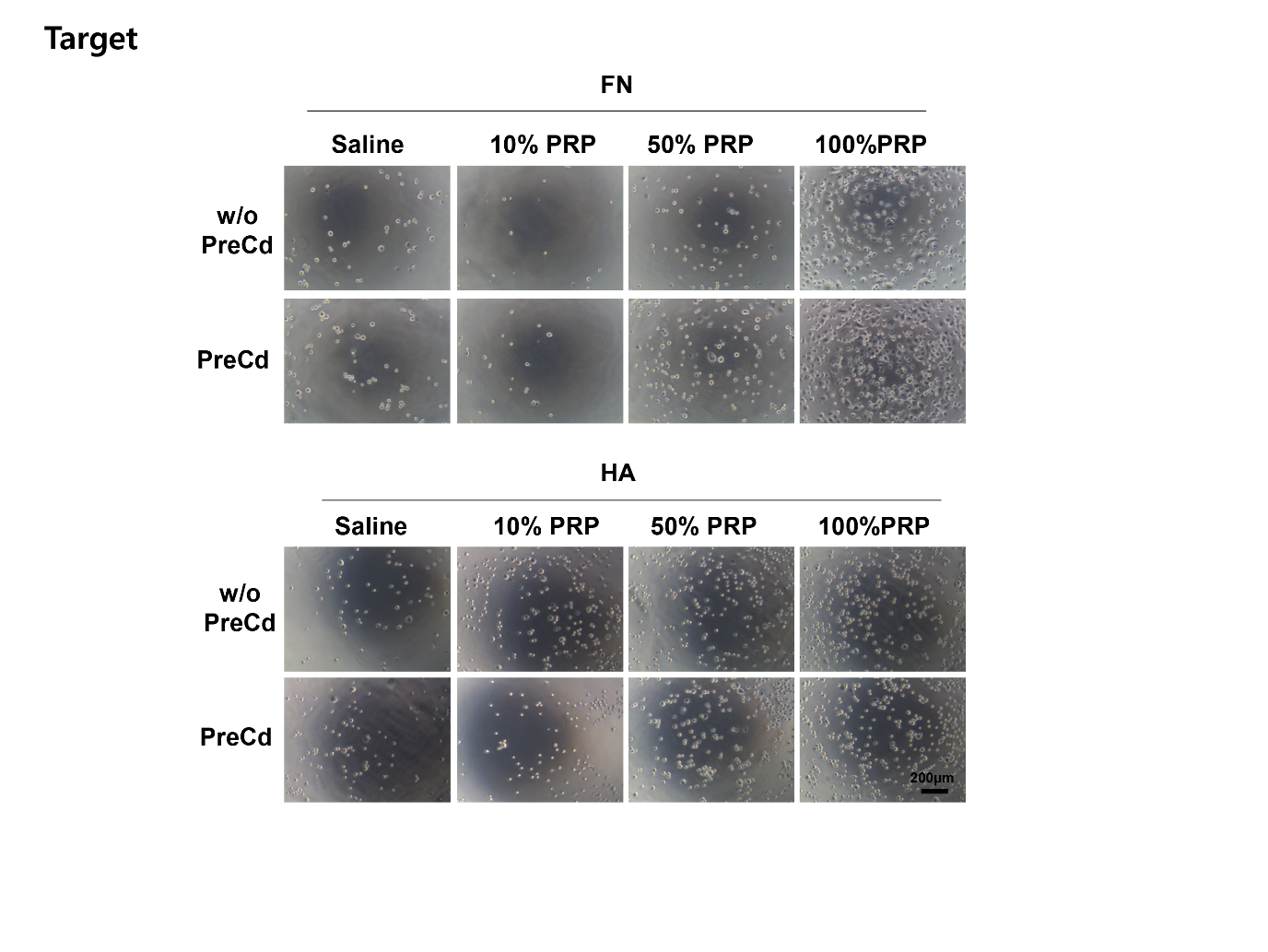


Supplementary figure 5. Representative images of adherent MSCs on FN and HA following preconditioning of both MSCs and ECM.


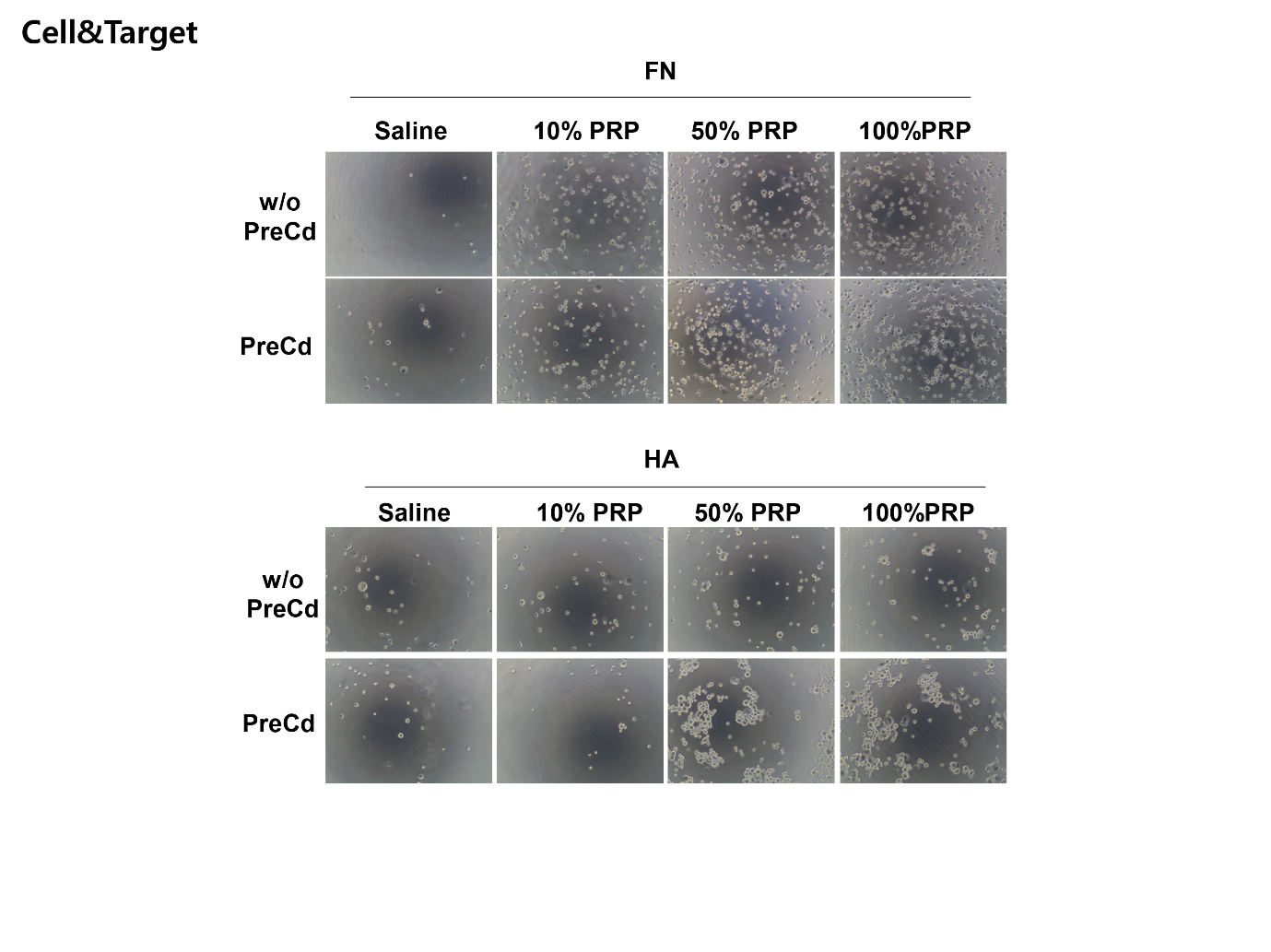
.
